# Equilibrated evolution of the mixed auto-/allopolyploid haplotype-resolved genome of the invasive hexaploid Prussian carp

**DOI:** 10.1038/s41467-022-31515-w

**Published:** 2022-07-14

**Authors:** Heiner Kuhl, Kang Du, Manfred Schartl, Lukáš Kalous, Matthias Stöck, Dunja K. Lamatsch

**Affiliations:** 1 Leibniz-Institute of Freshwater Ecology and Inland Fisheries—IGB (Forschungsverbund Berlin), Müggelseedamm 301, D-12587 Berlin, Germany; 2 University of Würzburg, Developmental Biochemistry, Biocenter, D-97074 Würzburg, Germany; 3 Xiphophorus Genetic Stock Center, Texas State University, San Marcos, TX 78666 USA; 4 Czech University of Life Sciences Prague, Prague, Czech Republic; 5 Amphibian Research Center, Hiroshima University, Higashi-Hiroshima, 739-8526 Japan; 6 Research Department for Limnology, Mondsee, University of Innsbruck, A-5310 Mondsee, Austria

**Keywords:** Genome evolution, Evolutionary genetics

## Abstract

Understanding genome evolution of polyploids requires dissection of their often highly similar subgenomes and haplotypes. Polyploid animal genome assemblies so far restricted homologous chromosomes to a ‘collapsed’ representation. Here, we sequenced the genome of the asexual Prussian carp, which is a close relative of the goldfish, and present a haplotype-resolved chromosome-scale assembly of a hexaploid animal. Genome-wide comparisons of the 150 chromosomes with those of two ancestral diploid cyprinids and the allotetraploid goldfish and common carp revealed the genomic structure, phylogeny and genome duplication history of its genome. It consists of 25 syntenic, homeologous chromosome groups and evolved by a recent autoploid addition to an allotetraploid ancestor. We show that de-polyploidization of the alloploid subgenomes on the individual gene level occurred in an equilibrated fashion. Analysis of the highly conserved actinopterygian gene set uncovered a subgenome dominance in duplicate gene loss of one ancestral chromosome set.

## Abstract (web summary)

The haplotype-resolved assembly of the asexual invasive Prussian carp shows six genome copies (AAABBB), evolved from two ancestral species by a recent self-addition (AB) to its hybrid-tetraploid (AABB) goldfish ancestor. Equilibrated gene loss led to subgenome dominance.

## Introduction

Polyploidy, the genetic condition where more than two sets of homologous chromosomes are present, is a widespread phenomenon that has been studied intensively since more than 100 years when it was first detected by DeVries^1^ and Blakeslee^2^. Its biological significance ranges from pathological conditions to being an important driver of evolution and a beneficial situation for crop plant breeding^3^. It is a widespread phenomenon in plants but much rarer in animals^4–6^. Major questions for understanding polyploidy include the molecular consequences of increased gene dosage, the impact on sex determination mechanisms^7^, meiosis and physiology as well as the interaction with their diploid congenitors^8^. With respect to reproduction, uneven levels of ploidy, e.g., triploidy, are particularly problematic because of the impact on meiosis. In consequence, triploid lineages generally escape this constraint by turning to various forms of asexuality^7,9^. One paradigmatic example is the diploid—polyploid complex taxonomically recognized as Prussian carp, *Carassius gibelio*, of which sexual and asexual biotypes of various ploidy have been described^10,11^. It emerged that the lineage of cyprinids to which the Prussian carp is assigned had an allotetraploid origin^12–14^^.^ Prussian carps usually inhabit freshwater benthic habitats of the Northern hemisphere of Eurasia and have recently been introduced into the United States^15^. In Europe, *C. gibelio* is the most successful invasive fish species. It has a high ecological and economic impact^16^, due to predation, competition, alteration of ecosystems, transmission of diseases and hybridization. It presents a threat to native aquatic vertebrates, especially for the endangered indigenous Crucian carp, *Carassius carassius*.

Asexual biotypes of *Carassius gibelio* produce unreduced eggs, whose embryogenesis is triggered by host-sperm to develop parthenogenetically (gynogenesis). They exploit various syntopic host-species including feral goldfish (*Carassius auratus*), Crucian carp (*Carassius carassiu*s) and common carp (*Cyprinus carpio*)^17^. Mostly, the paternal DNA is eliminated from the oocyte and the offspring is generated clonally. However, introgression of paternal DNA has been repeatedly described (for details of the reproductive complex see refs. ^18–20^ and references therein). Though much has been learned about the gynogenetic reproductive mode from laboratory-produced clones of the Prussian carp^21^, we still lack information on the genetics and genomics of this important freshwater species. Even the evolutionary origin of the natural polyploid forms has been unknown.

Genomic resources are necessary for understanding the physiological and ecological traits and sex determination system as well as for management of natural populations. Therefore, in this study we sequenced the genome of a polyploid female of *C. gibelio* from a wild population. The high-quality assembly of all 150 chromosomes uncovered that this biotype is indeed a naturally evolved hexaploid, interestingly combining features of allo- and autopolyploidy. We identified an autoploidy event as the origin of the asexual lineage and provide first insights into its evolution with respect to gene content and expression of all six subgenomes.

## Results

### Genome sequencing, chromosome assembly and annotation

**Fig. 1:**
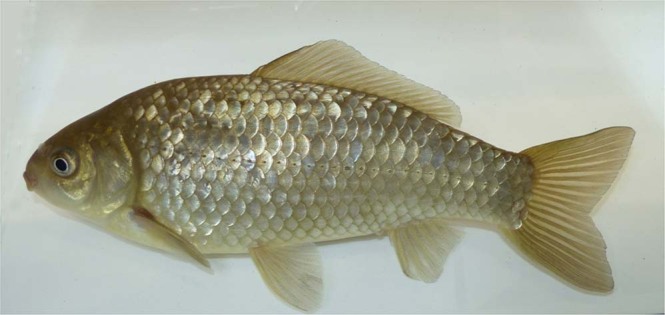
Female Prussian carp (*Carassius gibelio*). Image of an asexual hexaploid female from the source population.

To obtain a polyploid genome assembly of highest contiguity and completeness, we first sequenced the DNA from a single hexaploid female (Fig. 1) with HiFi PacBio technology at ~45-fold coverage per haplotype (which corresponds to a 135-fold genome-wide coverage if haplotypes would be collapsed) and an average read length of 19 kb (Supplementary Data 1a). Chromosome conformation capture (Hi-C) Illumina read pairs (*n* = 1,639,548,159, ~97-fold coverage per haplotype) from the same individual were generated. The PacBio reads were assembled into haplotigs using Hifiasm and the Hi-C reads were mapped on the haplotigs to produce scaffolds with a scaffold N50 of 30.7 Mb and contig N50 of 4.3 Mb. After manual curation we obtained 150 long scaffolds corresponding to the complete hexaploid chromosome number of the Prussian carp. In total 93% of the 5.07 Gb assembled sequences were assigned to chromosomes.

To annotate protein-coding genes, gene evidence from protein homology of other species, RNA-seq transcriptomes from the Prussian carp and ab initio predictions were integrated. A total of 152,308 protein-coding genes were annotated (Supplementary Data 2), of which 150,182 (98.6%) have a Blast hit to the Swissprot/Refseq database, and 130,496 (85.7%) to the Pfam database. In total, 9015 (5.9%) of the protein-coding genes are single exon genes. Additionally, 16,571 tRNA, 348 rRNA, 1495 miRNA, and 9258 other non-coding RNA (ncRNA) genes were annotated (Supplementary Data 3a). In total, 18,131 (12%) pseudogenes were predicted. The BUSCO completeness based on the Actinopterygii_odb9 dataset was improved from 94.7 to 99.2% by the annotation process (Supplementary Data 2).

The Prussian carp genome has a repeat content of 41.3% (Supplementary Data 3b), slightly higher than reported for common carp (39.2%) and goldfish (39.6%)^22^. The amount of interspersed repeat elements in the genome was 35.1% of which 5.9% were considered as unknown. The most abundant classes of transposable elements are Helitrons (3.19%), L2 (2.85%) and hAT-Ac elements (2.54%).

### Haplotype-resolved structure of the hexaploid genome

The assembly of all 150 chromosomes of the Prussian carp allowed us to assess the entire genomic structure, phylogeny and ploidy history of a polyploid animal. Alignment of the assembly to those of the sexually reproducing tetraploid goldfish, tetraploid Common carp, and three diploid cyprinids, the grass carp (*Ctenopharyngodon idella*) and *Onychostoma macrolepis*, and the zebrafish (*Danio rerio*) genomes identified 25 groups of widely syntenic, homeologous chromosomes.

**Fig. 2:**
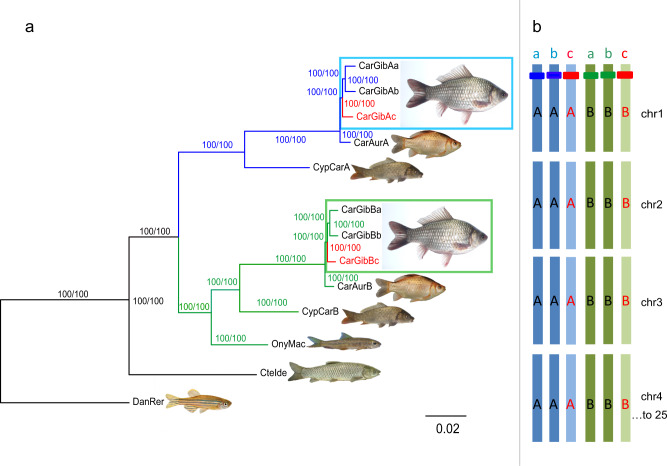
Phylogenetic relationships and subgenome structure. **a** Phylogenies using maximum likelihood (ML) based on chromosome-wide alignments (coding and non-coding sequence) of each of the 6 × 25 haplotypes of the *C. gibelio* hexaploid genome. Its haplotypes (CarGib_Aa, _Ab,_Ac, _Ba, _Bb, _Bc) are shown in comparison to collapsed chromosome-scale data of the ancestral diploid cyprinid grass carp (CteIde), and the diploid cyprinid *Onychostoma macrolepis* (OnyMac), allotetraploid common carp, *Cyprinus carpio* (CypCar), allotetraploid *Carassius auratus* (CarAur), and diploid zebrafish (DanRer). Note the slight difference in branch lengths for the subgenomes A vs. B. The tree was calculated from a genome-wide concatenation of the data analyzed for the 25 single trees and the addition of zebrafish as outgroup (in total 183.5 Mbp alignment length corresponding to 20.8% of the *Onychostoma macrolepis* reference chromosomes; gap content comprised 9.8%). Values for Shimodaira–Hasegawa approximate likelihood ratio tests and ultrafast bootstrap support (SH-aLRT [%]/UFB [%]) methods are given at each node, where 100/100 presents the percentage of bootstrap support. Note that due to methodology, Aa and Ab are collapsed to CypCar_A and CarAur_A as well as Ba and Bb are collapsed to CypCar_B and CarAur_B, respectively. **b** Subgenome structure of the formally hexaploid Prussian carp (*C. gibelio*) genome. The two subgenomes A and B, each with three haplotypes a, b, c (above), symbolize chromosome sets of the two diploid ancestral species (AaAb and BaBb, each with 25 chromosomes), which gave rise to the allotetraploid common ancestor (AaAbBaBb) of the *Carassius* and *Cyprinus* lineage by an ancient hybridization event. The Prussian carp arose by an autopolyploid addition of two chromosome sets (AcBc) and thus contains 150 (= 6 × 25) chromosomes. Blue or green (a, b) and red (c) bars represent homologous alleles (among homologous haplotypes a–c: blue or green) and homeologous alleles (between subgenomes A or B: blue *vs*. green).

Phylogenetic trees calculated for each of the syntenic chromosome groups (Fig. 2a and Supplementary Fig. 1) revealed a structure of the Prussian carp genome to be composed of three groups of haplotypes (a–c, Fig. 2b) each of which consisting of two haplotypes made up by the chromosomes representing the allotetraploid structure of the carp and goldfish cyprinid genomes, designated A and B (i.e., AaAbBaBb), respectively. This generates a total of two triplicates similar to A and B, i.e., six haplotypes (AaAbAcBaBbBc), each with 25 chromosomes. In the chromosomal trees, for most ancestral cyprinid pair of chromosomes (1–25) across the six haplotypes, the one from the AcBc set was phylogenetically separated (100% bootstrap support in most cases) from the two others and more closely related to *C. auratus* (Fig. 2a). We thus assigned these and the remaining chromosomes to haplotype c, if at least two of the three analyses (12 taxon tree, 4 taxon tree, and divergence estimates) supported the respective chromosomes being the outgroup of the three haplotypes (Supplementary Data 4a–d). These were unambiguously assigned to haplotype c, while assignment to haplotypes a and b remained arbitrary.

**Fig. 3:**
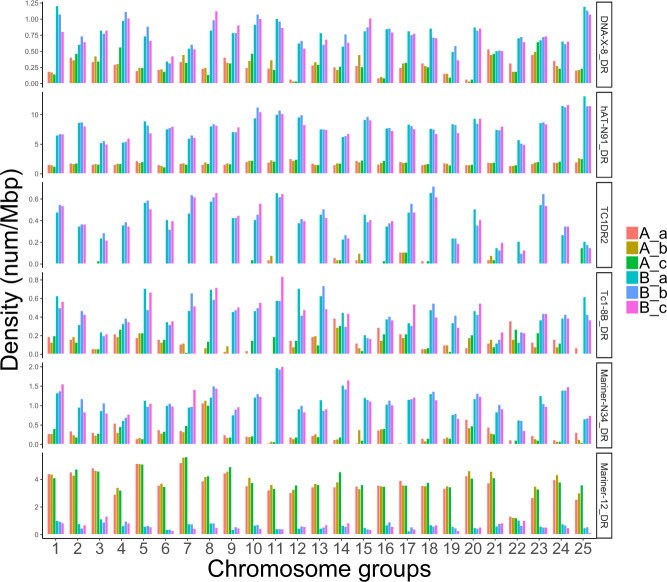
Density of transposable elements DNA-X-8_DR, hAT-N91_DR, TC1DR2 and Mariner-12_DR on chromosomes of the hexaploid Prussian carp showing A and B subgenome-biased distributions. The density of TEs was calculated as the frequency (num) normalized by chromosome length (Mbp).

To elucidate the origin of the three subsets of corresponding chromosomes (i.e., haplotypes a, b, c) by auto- or allopolyploidy, we applied a strategy that has been successfully used to show the allopolyploid structure of the *Xenopus laevis* genome^23^ and the autopolyploidy of the sterlet sturgeon^24^. It is based on the reasoning that in case of allopolyploidy the fast-evolving repeats and relics of mobile elements are specific to the allopolyploid ancestors and thus would cluster in separate branches of phylogenetic trees. For an autoploid chromosome set the repeat elements would not differentiate the homeologs. For the goldfish genome the carp-specific whole genome duplication (Cs4R) was confirmed as allopolyploidy based on 20 types of TEs^13^ that are almost exclusively enriched in one subgenome. Using these 20 TEs and five additional ones detected by the SBI analyses (see next paragraph) we find that in the Prussian carp genome individual TEs are enriched for either one of the A or B subgenomes in accordance with the allopolyploid history (Cs4R) of a tetraploid cyprinid ancestor (Fig. 3 and Supplementary Fig. 2). However, the three haplotypes a, b, and c did not significantly differ in TE density, pointing to an autopolyploid origin.

**Fig. 4:**
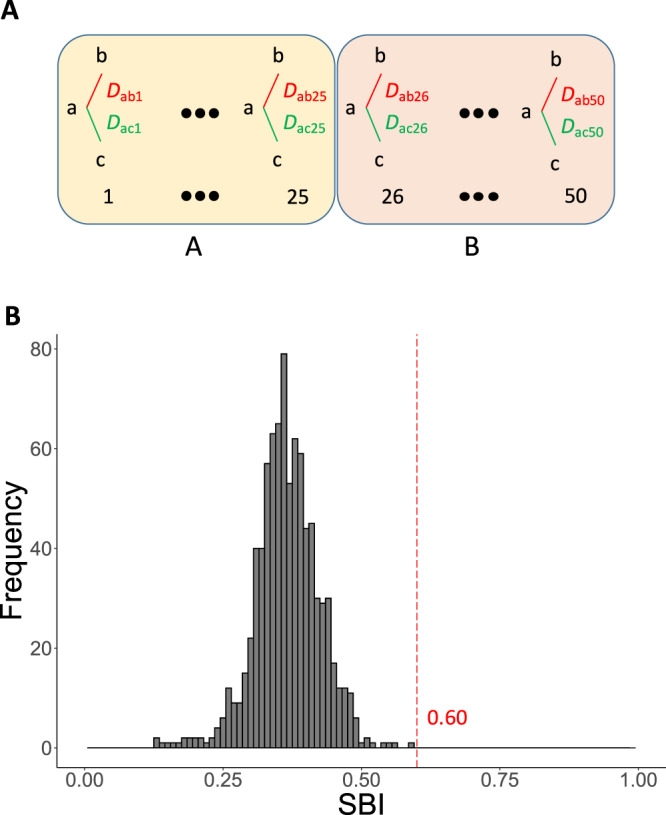
Calculation and distribution of subgenome-biased index (SBI) for TEs in the reference genome of the hexaploid Prussian carp. **A** The scheme used to calculate the SBI for each TE according to the formula $SBI=\left\{\mathop{\sum }\limits_{i =1}^{50}|{{{{D}}}}_{{{{{{\rm{ab}}}}}}i}-{{{{D}}}}_{{{{{{\rm{ac}}}}}}i}|\right\}/\left\{\mathop{\sum }\limits_{i=1}^{50}({{{{D}}}}_{{{{{{\rm{ab}}}}}}i}+{{{{D}}}}_{{{{{{\rm{ac}}}}}}i})\right\}$ where D_ab_ represents the absolute difference of frequency of the TE on chromosome a and b, and D_ac_ between chromosome a and c. **B** The distribution of SBIs of TEs in the reference genome.

In another approach to uncover if the latest polyploidy event of Prussian carp was an allo- or autopolyploidization, we adapted and re-defined the subgenome-biased index (SBI)^13^. The SBI-value of a TE reaches from 0 to 1, with the value close to 1 indicating the TE type is enriched differentially in one of the chromosomes from the a, b and c triplet. Such TEs could be used to tell apart allelic from ohnologous chromosomes, and to distinguish allo- or autopolyploidization of the most recent elevation of ploidy generating the hexaploid Prussian carp. However, no such TEs were evident from this analysis (Fig. 4B and Supplementary Fig. 3), hence providing no evidence for allopolyploidy of the a, b, and c haplotypes.

The three haplotypes a, b, and c, which according to all analyses above have an autoploid relationship, show divergence values for all chromosomes in the range of 1% (Supplementary Data 4a). Such difference could be due to accumulation of mutations between originally identical endoautoploid chromosome sets or reflect the genetic intraspecific divergence of chromosome sets from an ancestral tetraploid Prussian carp (e.g., Aa, Ba, Ab, Bb) and the donor of the two additional haplotypes (e.g., Ac, Bc) that generated the hexaploid gynogenetic lineage. We roughly estimated the intraspecific genetic divergence by comparing our genome assembly from a European fish to the collapsed genome assembly of a Prussian carp from China^19^. This revealed a similar divergence value as for the three chromosome sets to each other, with haplotype c being marginally closer to the fish from China than haplotypes a and b (Supplementary Data 5).

In the goldfish, the anti-Müllerian hormone encoding *amh* gene was identified as the potential male sex determining gene on chromosome 22 from the B genome but not on the alloploid homeologue from A^25^. Interestingly, we also found *amh* only on Prussian carp chromosomes 22Ba, 22Bb and 22Bc. Of note, other genes known to be involved in sex determination, e.g., gonadal soma derived growth factor, *gsdf*, or doublesex and mab-3 related transcription factor 1, *dmrt1*, have the expected six copies in the Prussian carp genome (Supplementary Fig. 4).

### Phylogenomics and divergence time estimates

**Fig. 5:**
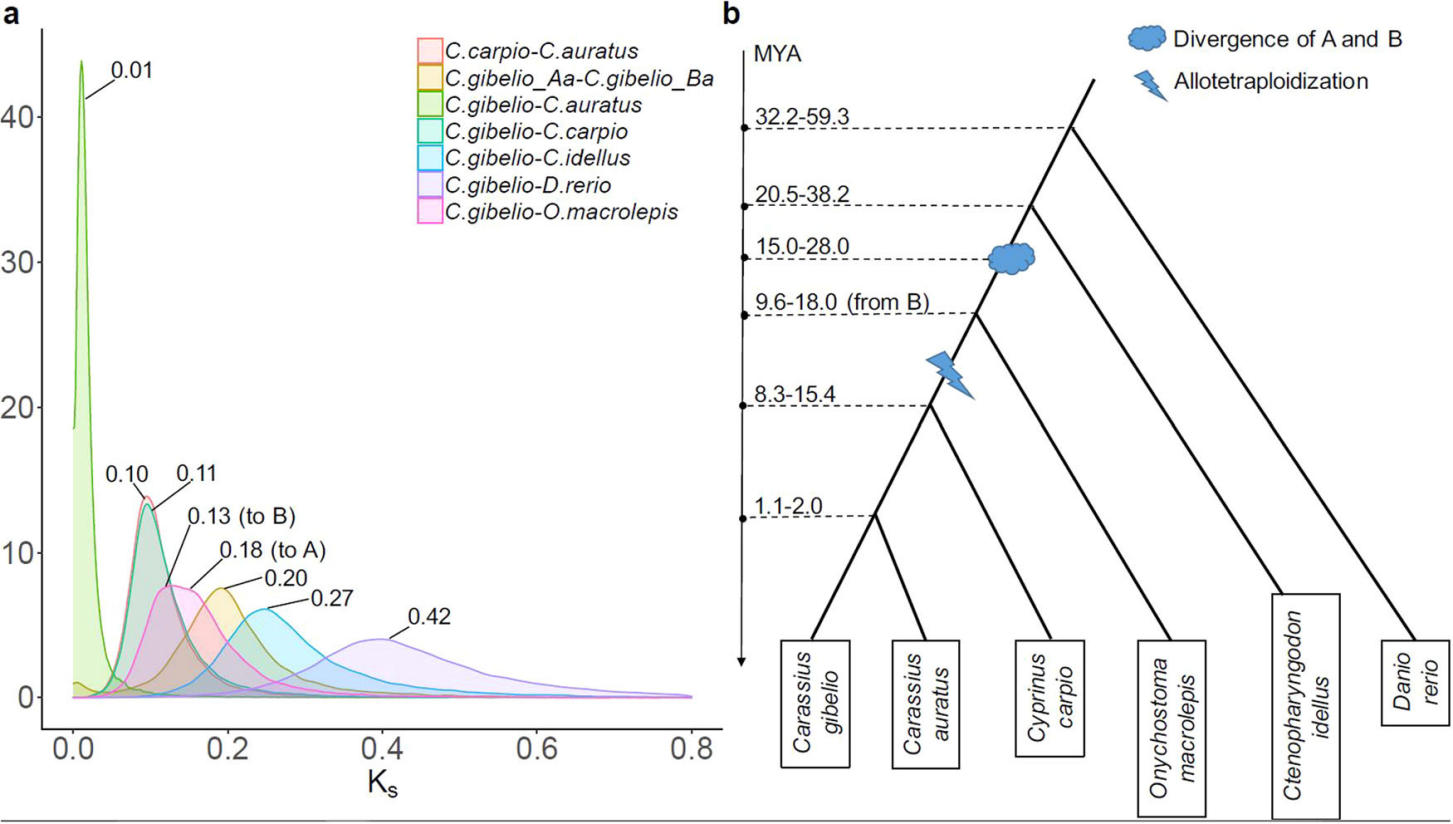
Molecular dating of species’ divergences and polyploidization events. **a** Distribution of synonymous substitution rates (Ks) between species and between subgenomes A and B in the Prussian carp, *C. gibelio*. Numbers at distribution peaks indicate median Ks values. **b** Time estimates of species' divergences and the common allotetraploidization event of the *Carassius* and *Cyprinus* lineage.

To determine the phylogenetic position of the Prussian carp within the Cyprininae and estimate the timing of divergence between species, the orthology gene set from all species and the A and B subgenomes of *C. gibelio* was established and the synonymous substitution rate (Ks) for genome and subgenome pairs determined (Supplementary Data 6). For calibration served the consensus Ks-derived divergence time range from four independent studies^12,22,26^. In accordance with previous mitochondrial sequence and single gene data^15,27^ *C. gibelio* is most closely related to the goldfish. Both lineages diverged only between one and two million years ago. Adding the Prussian carp to the phylogenomic tree confirmed also the much longer ancestral divergence time of the A and B genomes in the ancestors of the allotetraploid *Carassius* lineage at about 15–28 Mya. The tetraploidization event is estimated between 8.3 and 18 MYA (Fig. 5).

### Subgenome dominance and rediploidization

Genome evolution of the hexaploid *C. gibelio* comprises two distinct phases: the evolution of the ancient subgenomes A and B from the allotetraploid cyprinid ancestor and the autoploid addition of the AcBc-complement.

The process of depolyploidization^28^ in this allopolyploid lineage (here A and B subgenomes), which finally results in the loss of one of the duplicates, may either be random and loss occurs on both subgenomes or it can take place preferentially in one subgenome. Like in the common carp^29^ the total number of protein-coding genes of subgenome B (BaBbBc = 77,204) of the Prussian carp is slightly higher than that of the A subgenome (AaAbAc = 75,104) (Supplementary Data 2). This difference in gene content can be explained by the greater length of the B sets of chromosomes 3 and 22 (Supplementary Fig. 5). However, signs of differential depolyploidization between subgenomes A and B became apparent when both subgenomes were inspected for the subset of 4584 genes, which are highly conserved in all actinopterygian fish, as listed in the BUSCO catalog (Supplementary Data 7a).

In the—according to branch lengths—faster evolving subgenome A 12.8–13.4% of BUSCO genes are missing, while subgenome B has lost only about 7.8–8.2% (Supplementary Data 7a). Similar as in Prussian carp, the goldfish subgenome A has lost about 5.2% more BUSCO genes than subgenome B. Analyzing the BUSCO gene set in the *C. carpio* subgenomes A or B (GCF_018340385.1^30^) generated similar results with 12.1% of genes missing in A and 9.2% in B, i.e., a difference of 2.9%. Regarding overlap of BUSCO genes, there are 444 identical BUSCOs missing in the subgenomes A of both, *C. gibelio* (all haplotypes) and *C. auratus*, but they are present in subgenome B of both species (Supplementary Data 7b). In contrast, we find 235 missing BUSCOs that are specifically lost in subgenome B of both species. When adding common carp to this analysis, we still find subgenome-specific gene loss happening in all three species for 266 and 133 BUSCOs in subgenome A or B, respectively. Thus, the ratio of subgenomic BUSCO gene loss (subgenome A/B) ranged from 1.9 to 2.0. Functional enrichment analysis of the BUSCO genes subject to depolyploidization revealed strong enrichment of GO terms for nc/rRNA related processes and DNA recombination, replication and repair for all of the different combinations of species overlaps (Supplementary Data 7c). Interestingly, combined BUSCO scores of A + B suggested complementarity of the subgenomes since the number of missing BUSCOs remained very low (missing 0.6% in *C. gibelio* and 0.9% in *C. carpio*). Indeed, duplicates missing from the B subgenome are retained in A and vice versa. (Supplementary Data 7a).

The subgenome dominance of B is also supported by the phylogenomic analyses of the chromosome-wide alignments which revealed slight differences in the evolutionary speed as reflected by the branch lengths of the A and B subgenomes leading to the last common ancestor of *Cyprinus* and *Carassius* that evolved after the common allotetraploidization event (Fig. 2a).

Regarding the recent genomic autopolyploid genome addition (AcBc) of *C. gibelio*, we also analyzed BUSCO scoring differences by comparing the haplotype groups a, b and c. However, no significant differences between haplotypes a–c were detectable with differences between missing BUSCOs only ranging between 0.2–0.3%. These low differences might be skewed due to annotation or assembly issues. Thus, we analyzed only genes that were missing in haplotypes a–c (BUSCO and annotation) and also checked for haploid sequencing coverage of their remaining alleles to identify genes (Supplementary Data 8) putatively deleted after the Prussian carp autopolyploidization. Of 24 genes, three are related to GO term “chromosome organization” (*kansl3, mapk15, ighmbp2*) and one gene is related to “regulation of mitotic cell cycle“ (*sra1*). This low number of putatively deleted genes may be due to the short time that passed since the Prussian carp emerged. Our results are in accordance with findings of selection acting on DNA repair/management/meiosis genes after recent polyploidizations of plants^31^.

A similar picture emerged for the microRNAs and the group of “other” ncRNAs, which show no biased frequency for certain chromosomes across all six haplotypes (Supplementary Fig. 6). Conversely, tRNA and rRNA gene frequency has a clear bias towards certain chromosomes. Interestingly, this was not always confined to either the A or B subgenomes, but for instance in the case of chromosome 3, rRNAs are encoded in haplotypes Ac, Bb and Bc but not in the three other haplotypes. On the other hand, rRNA loci on chromosome set 7 are only encoded on the A derived subset. tRNA genes are enriched on the B subset of chromosome 6 or on A of chromosomes 8 and 9. Chromosome 17 is enriched for tRNA genes only on chromosome 17Aa and 17Ab but not on 17Ac.

**Fig. 6:**
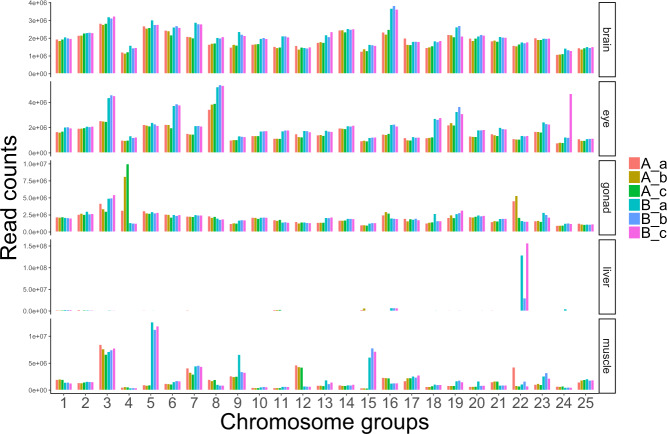
Counts of RNA-seq reads from different organs mapped on the 150 chromosomes of *C. gibelio*. Chromosomes are arranged in 25 syntenic groups, ordered from left to right as subgenomes and haplotypes AaAbAcBaBbBc. A similar result was obtained when only uniquely mapping reads were used.

In allopolyploid genomes, which feature subgenome dominance with respect to duplicate loss, overexpression of the genes from one of the parental subgenomes also occurs. Thus, we compared transcriptional subgenome activity by mapping the RNA-seq reads to each haploid chromosome (Fig. 6). This revealed no general dominance of a certain allopolyploid (A or B) subgenome or haplotype (a, b, c). Moreover, if preferential expression occurs, it is organ-specific and concerns only a few chromosomes. There is a tendency of increased gene expression toward the B subgenome (which has also lost less BUSCO genes), but chromosomes 4 and 22 from subgenome A show higher expression in the gonads. In a few cases one of the chromosomes from haplotypes a, b or c has an expression bias, e.g., chromosome 24Bb in the eye or chromosome 22Ba and 22Bc in the liver. Remarkably, this expression in the liver is about three orders of magnitude higher than from any other chromosome of the Prussian carp. A comparison of the expression of homeologous genes revealed that many gene pairs are preferentially expressed from either the A or B subgenome or one or two of the haplotypes, but overall there is no expression dominance of one subgenome or haplotype over the other (Supplementary Figs. 7 and 8).

## Discussion

Using long-read sequencing technology for deciphering the genome of the asexual Prussian carp we present here the first haplotype-resolved chromosome-scale assembly of a hexaploid animal. So far, polyploid animal reference genome assemblies had to collapse the homologous copies of every chromosome and thus form a “mosaic” reference of both parental haplotypes as a “monoploid” representation of the genome. Available genome assemblies of tetraploid vertebrates, such as African clawed frog (*Xenopus laevis*)^23^, common carp (*Cyprinus carpio*)^14^, and goldfish (*Carassius auratus*)^12,13,22,32^^,^ have resolved the alloploid subgenomes; however, the sequences of each of the homologs were shrunk into a single one. In polyploid eukaryotes, fine-scale haplotype assemblies have so far been computed in crop-species such as allohexaploid wheat^33,34^^,^ allotetraploid cotton^35^ or auto(formally octo-)polyploid sugarcane^36^. However, even in these prominent cases, the haplotypes were reconstructed by several direct and indirect methodologies, including BAC clones^36^, and expression data^37^ or were collapsed to monoploid haplotypes for each subgenome for annotation and data mining^38^.

In contrast, for the present *C. gibelio* assembly we made exclusively use of HiFi PacBio long reads, assisted by the Omni-C chromosome conformation capture technique, avoiding artifacts of homologous haplotypes to be wrongly forced into a single consensus^39^. The ordering of haplotigs directly into chromosome-level haplotype assemblies allowed to resolve all 150 chromosomes of the karyotype at comparable or even higher quality parameter scores as previous long-read scaffold-level collapsed assemblies of Prussian carp (NCBI accession GCA_019843895.1) or of the closely related tetraploid goldfish (Supplementary Data 1b).

Our haplotype-resolved assembly of the *C. gibelio* genome allowed us to reliably and more precisely place the Prussian carp in the genus and cyprinid family phylogenetic trees and to estimate its divergence from the other species with available genomes. The very recent divergence of goldfish and Prussian carp indicates a long common history and thus coevolution of their A and B subgenomes in a common allotetraploid ancestor. We noted that our phylogeny estimates a younger divergence of the zebrafish from the carp than previous studies^40^. This may be explained by lineage-specific differences in evolutionary rates/speed of the molecular clock between the rather short-lived zebrafish, which has a small body size, and the much bigger, long- lived carps. Body size is known to affect the speed of the molecular clock^41^, and generation time has also to be considered for divergence time estimates, suggesting that the more recent date for the zebrafish split may be more accurate.

The resolution of the chromosomal assembly on the level of all six haplotypes clearly reflects the allotetraploid origin of the evolutionary lineage leading to the present-day Prussian carps, represented by the A and B subgenomes. The ploidy elevation in the hexaploid Prussian carps was according to all analyses used here an autoploidy event. One more set of A and B subgenomes was added to an allotetraploid ancestor. Based on the divergence of the a, b, and c haplotypes, this was most likely not an endo-autoploidy, but an autopolyploid addition from another individuum of the same species (from the same or a different population). The somewhat closer relationship of the a and b haplotypes and the clustering of haplotype c with the corresponding chromosomes of goldfish as outgroup may suggest that haplotype c is the added one which gave rise to the gynogenetic biotype (Fig. 2). While the resulting allopolyploid Prussian carp is formally a hexaploid, its very special genomic composition is actually that of a classical imbalanced triploid (here consisting of strongly differing, deeply diverged triplicates AaAbAc and BaBbBc) facing the insurmountable problem of equally distributing (two times) three chromosome sets in meiosis, presumably causing its clonality.

Ploidy elevation above the default diploid state initially creates a “genomic shock”. For autopolyploidization it is reasoned to affect nucleotide pool imbalance^42^ and (usually prohibitive) meiotic difficulties encountered by young autopolyploids^31^. Allopolyploids face in addition the greater divergence and thus accumulated differences between the contributing genomes from distant organisms^7,43^. The resulting genomic conflict is hypothesized to be resolved by subgenome dominance and depolyploidization^44^. Importantly, recent research in polyploid plants suggests that one subgenome in allopolyploids may form most of the phenotype, methylation of transposons near genes is associated with subgenome dominance, and genetic incompatibility among subgenomes also causes such dominance patterns^45^. Comparing the different *C. gibelio* subgenomes, we can distinguish two evolutionary time periods: First, the evolution of the ancient allotetraploid cyprinid ancestor (exemplified by extant carp^14^ and goldfish^13^) leading to unequal but complementary evolution of subgenomes AaAb, and BaBb with evidence from slightly unequal branch lengths and differing retention of BUSCO gene duplicates. Such unequal gene content of the different subgenomes would indicate one subgenome to be preferentially subject to gene loss as a route to depolyploidization as described also for the African clawed frog (*X. laevis*) genome^23^. In contrast to the situation in *X. laevis*, in the allotetraploid common carp and goldfish, an almost equal representation of coding and non-coding genes and gene densities in the subgenomes have been reported^22^, in agreement with what we find in the Prussian carp. The overall view on the genome obviously camouflages that subsets of genes undergo duplicate loss, preferentially in one subgenome, as exemplified by the group of BUSCO genes. This loss of such a large number of BUSCO genes appears unlikely to have occurred in the diploid parental lineages, prior to the cyprinid allotetraploidization, since their status as being highly conserved amongst all actinopterygians indicates crucial functions in development and physiology and hence a higher selective pressure to return to regular diploid state. It may indicate that the previous view of a total structural parallelism of both subgenomes cannot be applied on the level of certain groups of genes. Support for a difference in subgenome evolution comes from our phylogenomic analysis based on chromosome-wide alignments, which revealed differences in the evolutionary speed as reflected by the branch lengths of the A and B subgenomes since their split from the last common ancestor. Enrichment analysis of the BUSCO genes subject to depolyploidization revealed that duplicates of genes involved in DNA recombination, replication and repair are preferentially lost. Interestingly, the same preference was also noted for the re-diplodization process in the sterlet sturgeon^24^ and even in plants^46^. This may be interpreted as genes and their gene products involved in these processes are adapted for a function in the singleton state.

Interestingly, we found that BUSCO genes lost in subgenome A are present with high probability in subgenome B and vice versa, supporting an “equilibrated” subgenome evolution.

A second period of subgenome evolution is the rise of allohexaploid *C. gibelio* by autopolyploid addition of the AcBc-complement. However, not enough time may have elapsed to detect gene duplicate loss.

With respect to gene expression the Prussian carp displayed very obvious instances of bias toward certain homologous or homeologous chromosomes. However, this was in no case consistently a feature of a whole auto- or alloploid subgenome, but appears to be a result of selection (rDNA, e.g., NOR silencing^47^ and possibly incompatibility, e.g., sex chromosome 22^7^,) affecting only some but specific chromosome groups. For instance, genes related to GO term rRNA are enriched in those that are lost in subgenome B, while all three A haplotype chromosomes 7 show a remarkable overexpression of rRNA genes. Chromosomes 4 and 22 from subgenome A have higher expression in the ovary than their B counterparts, and only one homeolog of chromosome group 24 is highly expressed in the eyes. Why genes with an obvious function for the development and or physiology of a specific organ have allele-biased or even allele-specific expression and understanding the underlying molecular mechanism, possibly related to epigenetic processes, will be interesting tasks for the future. In common carp and goldfish, expression of homeologous gene pairs showed biased B-subgenome expression^14^. It is known that epigenetic changes affect expression profiles. Thus, the difference between the two tetraploid sexual species and the hexaploid asexual Prussian carp may reflect epigenetic changes that are evoked by the higher ploidy level after addition of another set of the allopolyploid genome and/or their passage only through the female germline in the unisexual fish. To answer these questions, the genome and transcriptomes of an allotetraploid sexual Prussian carp have to be analyzed in the future.

Despite its asexuality, expected to accumulate deleterious mutations^48^, the mixed allo-/autohexaploid genome structure of the Prussian carp might cause its extremely high adaptability and broad ecological niche in Eurasian freshwaters, making it a successful invader^16^. Genomes, pre-adapted to different environments, create the potential for adaptation to a wider range of environmental conditions, as similarly suggested for allopolyploid plants^49,50^ and other animals^3,51^.

So far, the origin of the asexual lineage of the Prussian carp that turned out to be hexaploid and emerged from a tetraploid ancestor, was unclear and even an allopolyploid origin of the added chromosome set was discussed^52^. Our genome analyses do not support this and indicate an autopolyploid origin. The unique Prussian carp’s mixed allo- and autoploid evolution resulted in three deeply diverged homeologous A as well as three B subgenomes that long co-evolved in a tetraploid ancestor (Fig. 2), while the autopoplyploid AcBc-addition made it on the functional level a pseudotriploid asexual vertebrate.

## Methods

### Ethics and animal welfare statement

The research presented here complies with all relevant ethical regulations; naming the board/committee and institution that approved the study protocol. The fish was kept and sampled at the ILIM Mondsee in accordance with the regulations of the Austrian Animal Experiment Act (December 28, 2012) (Tierversuchsrechtsänderungsgesetz, part 1, section 1, §1, and point 2), and with the Directive 2010/63/EU of the European Parliament and of the Council of the European Union (September 22, 2010) on the protection of animals used for scientific purposes (chapter 1, article 1, and point 5a). The fish was kept according to regular aquaculture practice, including the provision of appropriate tank size, sufficient rate of waterflow, natural photoperiod, ad libitum food supply, as well as temperatures within the species’ thermal tolerance range. This ensured that no pain, suffering, distress or lasting harm was inflicted on the animal. Based on the legislative provisions above, no ethics approval and no IACUC protocol was required for sacrificing individuals after anesthesia with MS222 since it does not incur pain, suffering or distress to the fish, and no formal animal experimentation protocol was required.

### Experimental animals

Prussian carps were kindly provided by Jörg Bohlen and Lukáš Choleva (Institute of Animal Physiology and Genetics, Liběchov, Czech Republic). The fish used for this study originated from the Olsa river close to Ostrava (Czech Republic) because a *C. gibelio* neotype was described from this population^11^. Organs from a single female were sampled and flash frozen for further processing. Hexaploidy was confirmed by flow cytometry using DAPI, following the procedure described in ref. ^53^.

### Long-read library construction and DNA sequencing (HiFi)

HMW-DNA was extracted from ~25 mg snap-frozen tissue, stored at −80 °C until time of extraction, by tissueruptor homogenization according to Circulomics Nanobind Tissue Big DNA Kit Handbook v1.0 (https://www.circulomics.com/store/Nanobind-Tissue-Big-DNA-Kit-p129187130). Long-read libraries were produced using the SMRTbell Express Template Prep Kit 2.0 according to manufacturer’s instructions (Pacific Biosciences, Menlo Park, CA 94025). In brief, 15 µg of genomic DNA per library was sheared into 20 kb fragments using the Megaruptor 3 system. This was followed by removal of single stranded overhangs, DNA damage repair and end-repair/A-tailing before ligation of overhang hair-pin adapters to generate SMRTbell libraries. The libraries were then subjected to nuclease treatment using the SMRTbell Enzyme Clean Up Kit before size fractionation on the Sage Elf system according to the instructions from PacBio. Fractions 2 were selected for sequencing on the PacBio Sequel II instrument using the Sequel II Binding Kit 2.0, Sequel II Sequencing Plate 2.0 and the Sequel® II SMRT® Cell 8 M with 30 h movie time and 2 h pre-extension.

### RNA extraction

Total RNA was isolated using TRIzol Reagent (Thermo Fisher Scientific, Waltham, USA) according to the supplier’s recommendation, in combination with the RNeasy Mini Kit (Qiagen) from eyes, brain, liver, muscle and gonads from the same individual that was used for genome sequencing.

### TruSeq stranded mRNA sequencing

Library preparation with the Illumina TruSeq Stranded mRNA kit (Illumina Cat no: 20020595, Illumina, USA) included quality checks on total RNA; selective purification of mRNA from total RNA; synthesis of cDNA; adapter ligation followed by bead-based purifications (AMPure Beads).; amplification; quantitation of libraries for concentration estimates.

RNA concentration of was determined on the Qubit 3.0 (Thermo Fisher Scientific), and the quality of RNA based on the RNA Integrity Number was assessed on either the BioAnalyzer, Fragment Analyzer (Agilent), or Caliper GX instrument (PerkinElmer).

The subsequent steps of library preparation were all carried out on the Agilent NGS Bravo workstation in 96-well plates following the instructions for the Illumina TruSeq Stranded mRNA kit from Illumina. mRNA was purified from 300–700 ng of total RNA through selective-binding on poly dT-coated beads, and fragmented using divalent cations under elevated temperature.

Complementary DNA (cDNA) was synthesized from the resulting fragments by adding reverse transcriptase SuperScript II Reverse Transcriptase (Cat. no. 1808004 Thermo Fisher Scientific). The cDNA was then subjected to 3’-adenylation, which was followed by adapter ligation. The fragments with ligated adapters were amplified by PCR to amplify these fragments. The quality of the adapter-ligated libraries was checked on the BioAnalyzer or LabChip GX/HT DNA high sensitivity kit, and concentration by Quant-iT.

### Expression analyses

RNA-seq reads from brain, eye, gonad, liver and muscle were mapped on the genome using HISAT. The reads mapped on each chromosomes were counted using SAMTool^54^ and those mapped on genes were counted using featureCounts^55^. Transcripts per million of each gene were calculated using StringTie. The bar plots and heatmap figures were drawn using R^56^ with geom_bar in library ggplot2^57^ and heatmap.2 in gplots^58^, respectively.

### Dovetail Omni-C

Library preparation followed the “Nucleated Blood” section of the Dovetail protocol “Omni-C Proximity Ligation Assay, for non-mammalian samples, protocol version 1.0”. In brief, blood samples were defrosted and 10 µl of blood were crosslinked for 10 min in 3 mM disuccinimidyl glutarate, followed by an additional crosslinking of 10 min in 1% formaldehyde. The digestion conditions were determined by titration of the kit-provided Nuclease Enzyme Mix with final conditions of 6 µl of input blood digested with 0.5 units of Nuclease Enzyme Mix. Proximity ligation consisting of end-polishing, bridge ligation, intra-aggregate ligation and crosslink reversal using the Omni-C Kit from Dovetail was performed as described by the supplier. The chromatin was purified using AMPure XP beads (Beckman Coulter). In total, 150 ng of proximity ligated chromatin was used as input for library preparation using the “Dovetail Library Module for Illumina”. The libraries were subjected to 12 amplification cycles prior to DNA cleanup using AMPure XP beads. The final libraries were analyzed for fragment length and concentration using a bioanalyzer DNA high sensitivity chip and Qubit high sensitivity dsDNA, respectively, prior to sequencing on an Illumina NovaSeq 6000 sequencer in a 2x150 bp cycle setting.

### Haplotype resolved hexaploid genome assembly

Pacbio HiFi reads were assembled using Hifiasm (v0.15.1-r329^59^). The different resulting sets of contigs (“p_utg” and “p_ctg”) were checked for uniform coverage by remapping HiFi reads by Minimap2 (v2.22-r1101^60^) and calculating per base coverage using Bedtools genomecov (v2.25.0^61^). Multimodal distributions in coverage plots of “p_ctg” hinted at collapsed assemblies. The most uniform coverage distribution, with a single peak, was achieved by the “p_utg” set, which were considered fully resolved haplotigs (= a contig of reads that belong to the same haplotype). Additionally, the total consensus length of the haplotigs was approximately three times the size of *C. auratus* long-read assemblies available at NCBI (ranging from 1.6 Gbp to 1.8 Gbp, for details, Supplementary Data 1b).

Omni-C reads were mapped to the haplotigs by juicer (v1.5.7^62^). The resulting Hi-C links were filtered to remove links between allelic haplotigs by custom scripts. Filtered Hi-C links with mapping quality larger than 1 were then used for a first round of scaffolding by 3d-DNA (v180922^63^). Hi-C maps were inspected by Juicebox (v1.11.08^64^) and few apparent haplotig misassemblies were manually splitted. A second round of 3d-DNA scaffolding was performed. The Hi-C maps were inspected again by Juicebox and chromosomal scaffolds were manually assigned. Syntenic relationships of the scaffolds with *C. auratus* chromosomes were checked by whole genome alignment using minimap2 and plotted by Minidot (as part of Miniasm^65^). A final scaffolding step by 3d-DNA was applied separately on each chromosomal scaffold from the prior scaffolding step to improve contig ordering within the chromosomal scaffolds, which was confirmed by whole genome alignment to *C. auratus* genome (GCA_014332655.1^12^).

Final gap closure was performed by identifying end to start overlaps of neighboring contigs in scaffolds and by local re-assembly of reads mapping next to gaps as described in ref. ^66^. This is assembly version carGib1 and was used for assigning chromosome scaffolds to subgenomes and haplotypes.

### Phylogenetic trees of whole chromosomes and identity assignment

The hexaploid *Carassius gibelio* genome assembly was mapped by minimap2 to the following Cyprinidae genomes: diploid *Onychostoma macrolepis* (GCA_012432095.1^67^), diploid *Ctenopharyngodon idella* (GCA_019924925.1), allotetraploid *Cyprinus carpio* (GCF_018340385.1^30^), and allotetraploid *Carassius auratus* (GCA_014332655.1^12^). Syntenic chromosomes were identified and pairwise aligned by Last aligner (v941^68^) and 1-to-1 matches were filtered by Last-split^69^. Multiz (v11.2^70^) was used to combine all pairwise alignments into multiple alignments. Sequences that were aligned in all species were concatenated into a multiple fasta alignment by custom scripts. Phylogenetic trees were calculated per chromosome by Iqtree2^71^ using the specific best fit evolutionary model determined by Modeltest (as implemented in Iqtree2), for each of the 25 *C. gibelo* haplogroup alignments (i.e., chrA1 + chrB1…). This was repeated for a 4 taxon subtree consisting of only the chromosomes from the subgenome A or B (i.e. only chrA1 or only chrB1) of the *C. auratus* genome assembly and the corresponding three *C. gibelio* haplotypes to increase the number of alignable residues.

Additionally, sequence divergence between the multiple aligned haplotypes of *C. gibelio* was calculated.

Based on the 12-taxon trees, the 4-taxon trees and haplotype divergence, the chromosomes were named according to the *C. carpio* nomenclature (25 chromosomes per A and B subgenomes, https://www.ncbi.nlm.nih.gov/assembly/GCF_018340385.1) and suffixes a, b, c for the haplotypes were added. We assigned a chromosome to haplotype c, if at least two of the three analyses (12-taxon tree, 4-taxon tree and divergence estimates) supported the respective chromosome being the outgroup of the three haplotypes. In the few cases (<10%) where all three methods disagreed in the assignment of haplotype c, we used the tree with the best support values for assignment. If both trees contradicted each other, but had 100% support, we chose the 12-taxon tree to assign the c-haplotype. The assembly with reassigned c-haplotypes is carGib1.2.

### Genetic divergence among subgenomes and populations

To estimate the genetic divergence of populations and compare to that of subgenomes, we aligned the sequences of the three haplotypes (a, b and c) from our assembly of a European fish and with that of a fish from China^19^. The whole chromosome alignments were done using minimap2^60^, followed by chaining and netting^72^. The alignments were improved by adding alignments of flanked loci and TE (transposon element), removing obscure loci alignments and net filtering using GenomeAlignmentTool^73^. Then MULTIZ^70^ was used to combine pairwise alignments into a multiple alignment. Sequence difference was calculated as the percentage of SNP and indels.

### Repeat annotation

De novo repeat annotation was performed by running Repeatmodeler (v1.0.8^74,75^ on the whole genome. Repeat annotation was performed subsequently by Repeatmasker (version open-4.0.7^74^) using the de novo repeat library.

### Gene annotation

We performed gene annotation using two different approaches. In a comparative annotation approach, we splice aligned *C. auratus* Refseq. mRNAs (GCF_003368295) and proteins by Spaln (v2.06f^76^) to the *C. gibelio* assembly Cgi 1.0 separately for the three subgenomes (AB)a; (AB)b; (AB)c; and combined the resulting CDS and UTR matches into gene models by custom scripts. The second approach was based on a combination of homology and transcript evidence and ab initio prediction using previously described pipeline. For homology evidence, 645,452 protein sequences were used as query. These included the vertebrate database from Swiss-Prot (https://www.uniprot.org/), proteins with ID starting with “NP” from the RefSeq database (only “vertebrate_other”) and the human genome (GCF_000001405.39_GRCh38), all proteins from common carp (GCF_018340385.1), goldfish (GCF_003368295.1), zebrafish (GCF_000002035.6), platyfish (GCF_002775205.1), medaka (GCF_002234675.1), tonguesole (GCF_000523025.1), Mexican tetra (GCF_000372685.2) and northern pike (GCF_011004845.1). Genewise^77^ and Exonerate (https://www.ebi.ac.uk/about/vertebrate-genomics/software/exonerate) were used to map the query onto the repeat-masked assembly and to determine the gene structure. GenblastA^78^ was used prior to Genewise to find the rough alignment location. For transcriptome evidence, we collected the RNA-seq reads from eyes, brain, liver, muscle and gonads, and cleaned them using fastp^79^. Then we mapped the reads onto the repeat-masked assembly using HISAT^80^ and determined the gene structure using StringTie^81^. For ab initio prediction, Augustus^82^ was run with all predictions from above as hints and “--species = zebrafish” as the parameter.

To make the final consensus annotation, we screened homology gene models throughout the assembly: when multiple models compete for a splice side, the one with better support from StringTie wins; when a terminal exon (with start/stop codon) from ab initio or homology prediction is better supported by StringTie than the winner’s corresponding exon will be replaced. We also kept an ab initio prediction when its StringTie support was 100% and it had no homology prediction competing for splice sites.

The final annotation went through InterProscan (https://www.ebi.ac.uk/interpro/search/sequence/) for protein-domain check, BUSCO^83^ for assessing completeness, and Swissprot & Refseq blast^84,85^ for gene symbol and name assignment. In total, 120,475 (84%) of the 143,293 multi-exon gene had at least one splice site supported by RNA-seq data.

ncRNAs were annotated using the method adapted from Ensembl ((http://www.ensembl.org/info/genome/genebuild/ncrna.html)). tRNAscan-SE v.2.0.3^86^ was used for screening tRNAs, RNAmmer^87^ for ribosomal RNAs. microRNA and the remainder of the ncRNAs were predicted using Infernal with Rfam v.14.1^88,89^.

This generated annotation carGib1.2.gff and was used for genome-wide analyses. All gene annotations were benchmarked by BUSCO^83^ on each subgenome (Supplementary Fig. 9 and Supplementary Data 7a–c). Subsequently, the BUSCO analysis was focussed on missing genes in the subgenomes of *C. gibelio*, *C. auratus* and *Cyprinus carpio*. Venn diagrams for subgenome-specific missing BUSCO genes were plotted using the “Venn” online tool at https://bioinformatics.psb.ugent.be. Subgenome-specific missing BUSCO genes were analyzed for functional enrichment at www.mousemine.org.

### Subgenome bias index

For distinguishing auto- *vs.* alloploidy the subgenome bias index (SBI) was adapted from Kon et al.^13^ and re-defined. SBI was calculated as follows: for a given TE in each chromosome triplett (a, b and c of a chromosome in both subgenomes A and B), the absolute difference of its frequency of a to b, and of a to c were calculated as Dab and Dac respectively. Then the sum of the absolute difference between Dab and Dac was divided by the sum of their sum (Fig. 4A).

### Genome browser

A UCSC-like browser has been set up at http://genomes.igb-berlin.de^90^. The browser provides access to genomic sequences and annotations. A blat^91^ search tool is available for fast alignment of nucleotide or protein sequences against the genome.

### Dating of divergence

Divergence time of species and subgenomes was dated based on the Ks value (number of synonymous substitutions per synonymous site) as a molecular clock. Ks values were calculated between orthologs/ohnologs, which were identified using the reciprocal best blast hit method^84^, followed by strict synteny confirmation, where at least five orthologs/ohnologs had to be arranged in a row with the largest gap being fewer than five genes. Protein sequences of each pair of orthologs/ohnologs were aligned using MAFFT^92^ and then transformed into coding sequences (CDS) using PAL2NAL^93^. Pairwise Ks values were calculated based on the CDS alignments using codeml in PAML^94^. The median value of Ks of orthologous pairs was used to represent the divergence. The protein and coding sequences of the grass carp were downloaded from the GCGD database (http://bioinfo.ihb.ac.cn/gcgd/php/index.php)^95^.

### Reporting summary

Further information on research design is available in the Nature Research Reporting Summary linked to this article.

## Supplementary information

Supplementary Information

Description of Additional Supplementary Files

Supplementary Data 1-8

Supplementary Data 9

Supplementary Data 10

Supplementary Data 11

Supplementary Data 12

Supplementary Data 13

Supplementary Data 14

Supplementary Data 15

Supplementary Data 16

Reporting Summary

## Data Availability

All data generated in this study have been deposited in publicly accessible databases. Genome assembly, whole genome sequencing (WGS) data and RNA-seq reads are available under NCBI accession number PRJNA788516 and at the Prussian Carp genome browser http://genomes.igb-berlin.de/Prussiancarp/. The annotations are also available at the genome browser. Supplementary Data 9–16 present figure source files; for details see “Description of Additional Supplementary Files”.
