# Author Correction: Equilibrated evolution of the mixed auto-/allopolyploid haplotype-resolved genome of the invasive hexaploid Prussian carp

**DOI:** 10.1038/s41467-022-32470-2

**Published:** 2022-08-08

**Authors:** Heiner Kuhl, Kang Du, Manfred Schartl, Lukáš Kalous, Matthias Stöck, Dunja K. Lamatsch

**Affiliations:** 1 Leibniz-Institute of Freshwater Ecology and Inland Fisheries—IGB (Forschungsverbund Berlin), Müggelseedamm 301, D-12587 Berlin, Germany; 2 University of Würzburg, Developmental Biochemistry, Biocenter, D-97074 Würzburg, Germany; 3 Xiphophorus Genetic Stock Center, Texas State University, San Marcos, TX 78666 USA; 4 Czech University of Life Sciences Prague, Prague, Czech Republic; 5 Amphibian Research Center, Hiroshima University, Higashi-Hiroshima, 739-8526 Japan; 6 Research Department for Limnology, Mondsee, University of Innsbruck, A-5310 Mondsee, Austria

**Keywords:** Genome evolution, Evolutionary genetics

Correction to: *Nature Communications* 10.1038/s41467-022-31515-w, published online 14 July 2022

The original version of this Article contained an error in the Acknowledgements, which incorrectly read ‘We thank Maria Pichler for expert fish care and sample preparations, Wibke Kleiner and Simone Langone (ERASMUS+ trainee) for help in the RNA lab’. The correct version adds ‘and Josef Wanzenböck’ after ‘Maria Pichler’. This has been corrected in both the PDF and HTML versions of the Article.

