# Supplementary material for: Equilibrated evolution of the mixed auto-/allopolyploid haplotype-resolved genome of the invasive hexaploid Prussian carp
Source: Nat Commun. 2022 Jul 14;13:4092. doi: 10.1038/s41467-022-31515-w (PMC9283417; doi:10.1038/s41467-022-31515-w)
Supplement: Supplementary file 1 — Supplementary Information [file 41467_2022_31515_MOESM1_ESM.pdf]

Supplementary Information

Kuhl H. *et al.*

Equilibrated evolution of the mixed auto-/allopolyploid haplotype-resolved genome of the invasive hexaploid Prussian carp

Supplementary Figures

Supplementary Fig. 1 (next page): Phylogenies using Maximum Likelihood (ML) based on chromosome-wide alignments (coding and non-coding sequence) of each of the 6 x 25 haplotypes of the *C. gibelio* hexaploid genome. Its haplotypes (CarGib_Aa, _Ab,_Ac, _Ba, _Bb, _Bc) are shown in comparison to collapsed chromosome-scale data of the ancestral diploid cyprinid grass carp (CteIde), and the diploid cyprinid *Onychostoma macrolepis* (OnyMac), allotetraploid common carp, *Cyprinus carpio* (CypCar), allotetraploid *Carassius auratus* (CarAur), and diploid zebrafish (DanRer). Within the single chromosomal trees, *C. gibelio* subgenome designation (A or B) is followed by the chromosome number (1-25) and the haplotype designation (a-c). Note the slight difference in branch lengths for the subgenomes A *vs.* B. The tree in the center was calculated from a genome-wide concatenation of the data analyzed for the 25 single trees and the addition of zebrafish as outgroup (in total 183.5 Mbp alignment length corresponding to 20.8% of the *Onychostoma macrolepis* reference chromosomes; gap content comprised 9.8%). Values for Shimodaira–Hasegawa approximate likelihood ratio tests and ultrafast bootstrap support (SH-aLRT [%] / UFB [%]) methods are given at each node, where * presents 100% support. Note that due to methodology, Aa and Ab are collapsed to CypCar_A and CarAur_A as well as Ba and Bb are collapsed to CypCar_B and CarAur_B, respectively.

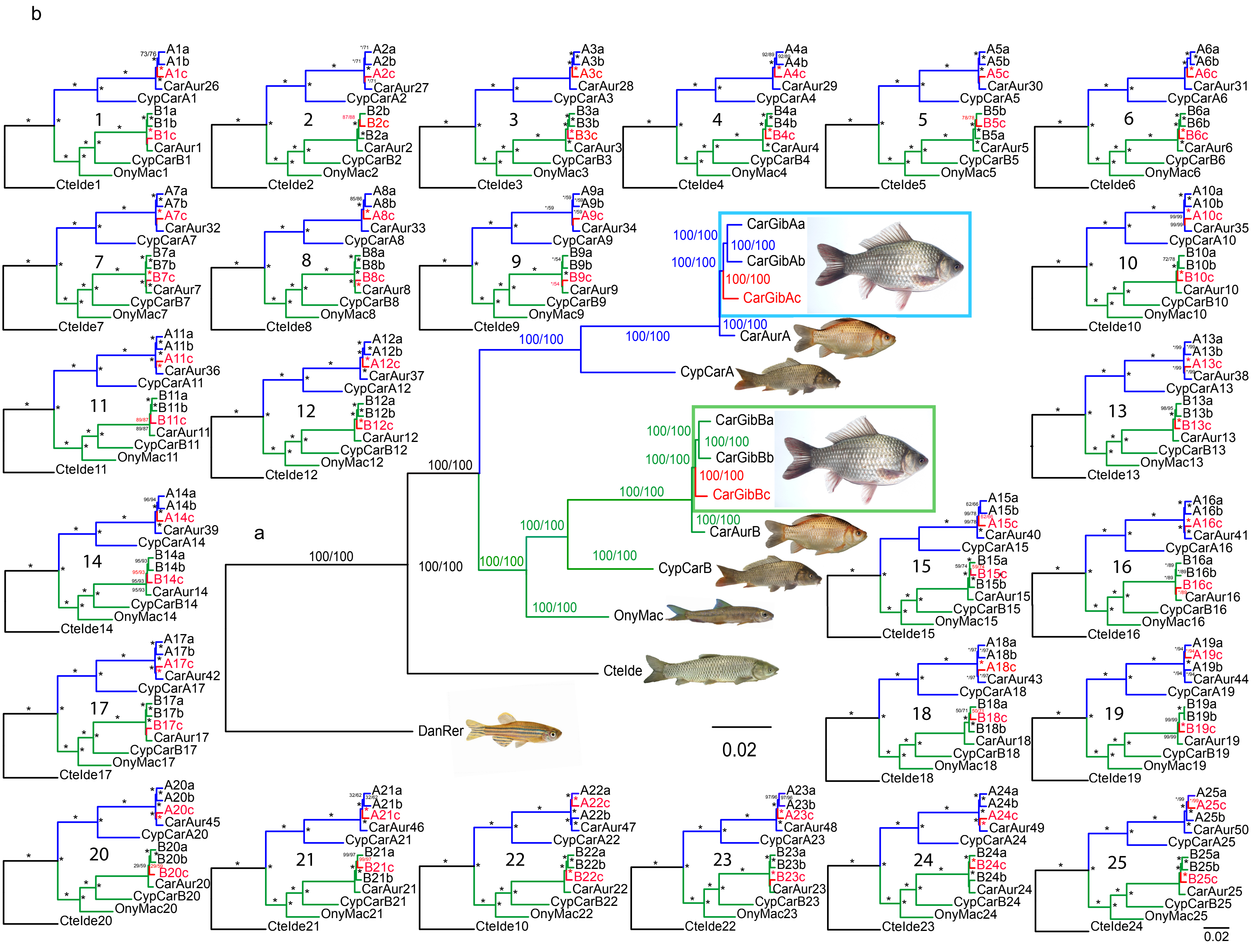

b
a
CarGibAa
CarGibAb
CarGibAc
CarAurA
CypCarA
CarGibBa
CarGibBb
CarGibBc
CarAurB
CypCarB
OnyMac
Ctelde
DanRer
100/100
0.02

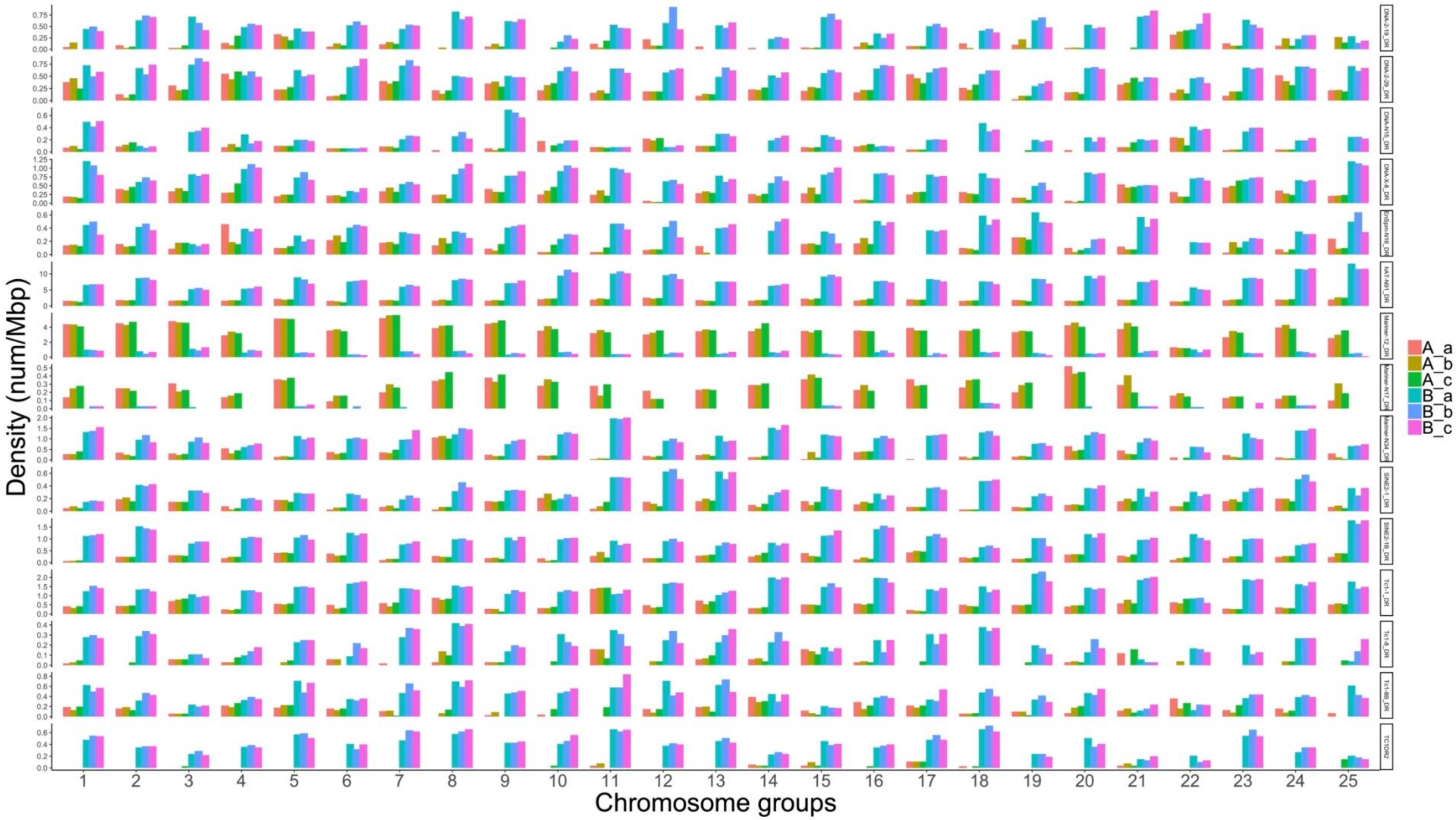

Supplementary Fig. 2: Density of 15 selected transposable elements on each chromosome.

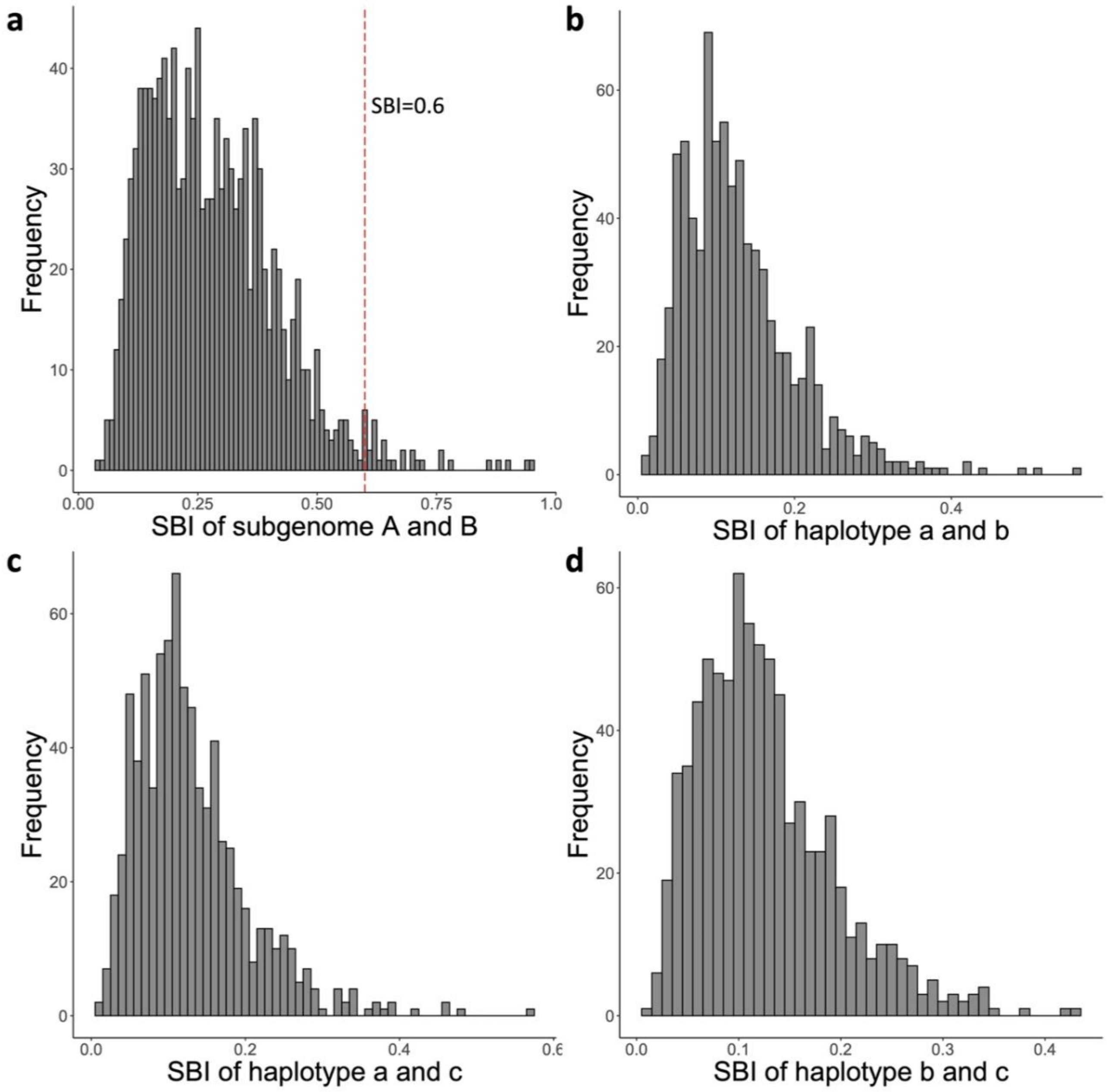

Supplementary Fig. 3: Distributions of subgenome-biased index (SBI). (a) SBI of subgenome A and B; (b) SBI of haplotype a and b; (c) SBI of haplotype a and c; (d) SBI of haplotype of b and c. The SBI is calculated using the formulas from a previous study[13].

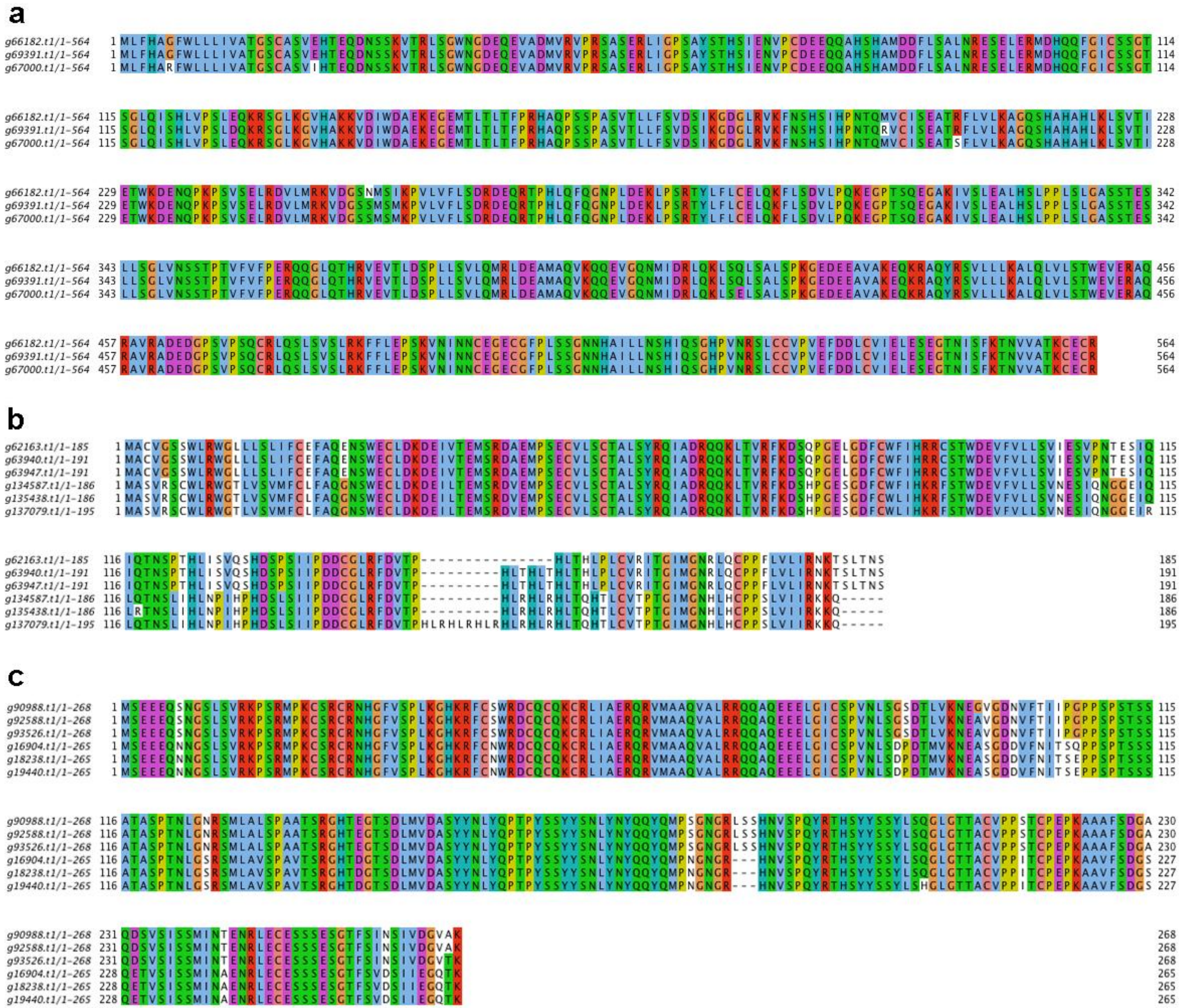

Supplementary Fig. 4: Protein sequence alignments of sex-related genes in Prussian carp. (A) Alignment of *Amh*. g66182.t1 is located on chromosome 22Bc; g69391.t1 22Bb; g67000.t1 22Ba. (B) Alignment of *Gsdf*. g62163.t1 is located on chromosome 21Ba; g63940.t1 21Bc; g63947.t1 21Bb; g134587.t1 21Ac; g135438.t1 21Aa and g137079.t1 Ab. (C) Alignment of *Dmrt1*. g90988.t1 is located on chromosome 5Ac; g92588.t1 5Aa; g93526.t1 5Ab; g16904.t1 5Bc; g18238.t1 5Ba and g19440.t1 5Bb.

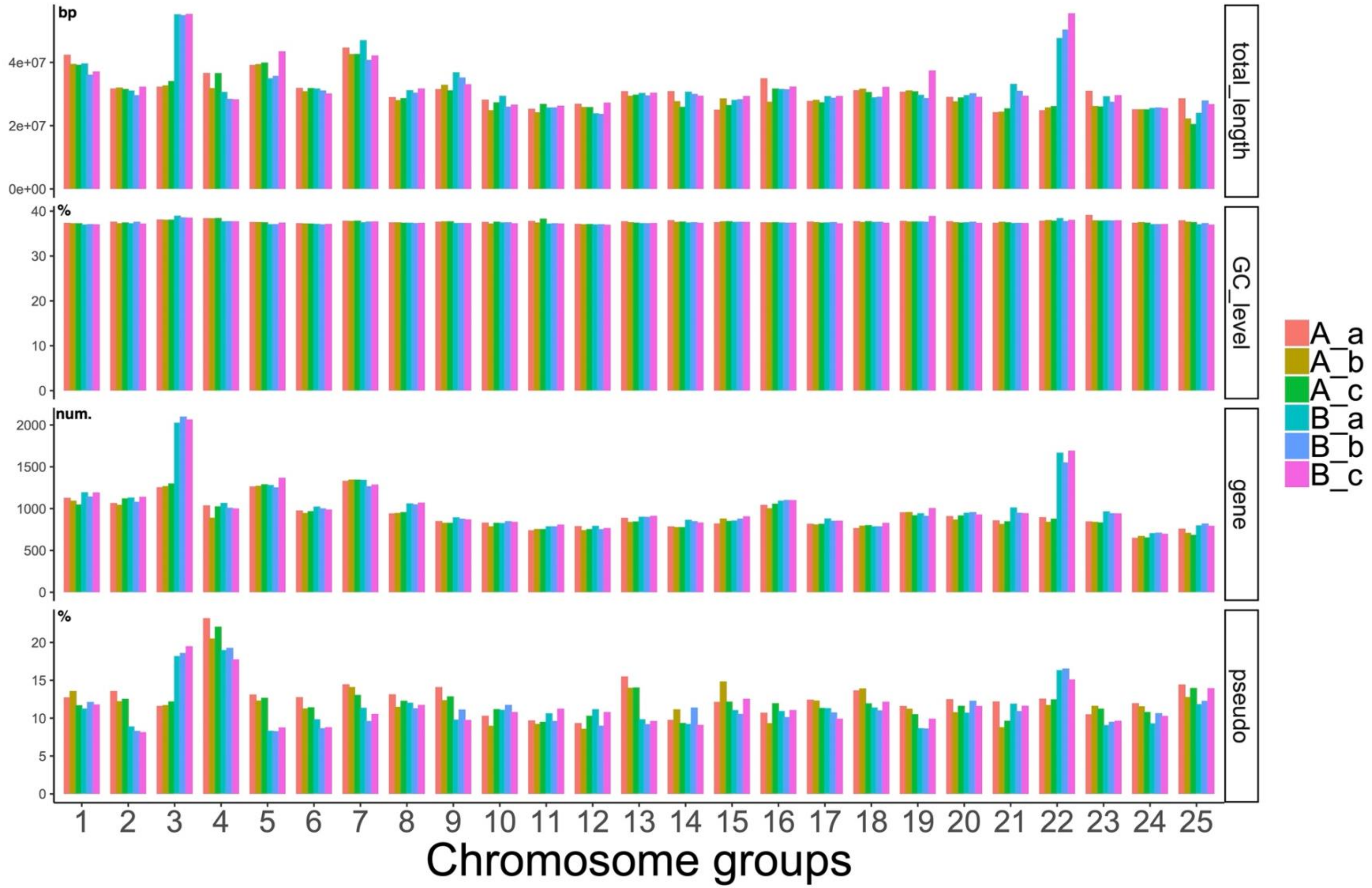

Supplementary Fig. 5: Total length, GC content, protein-coding gene frequency and the fraction of pseudogenes on each chromosome.

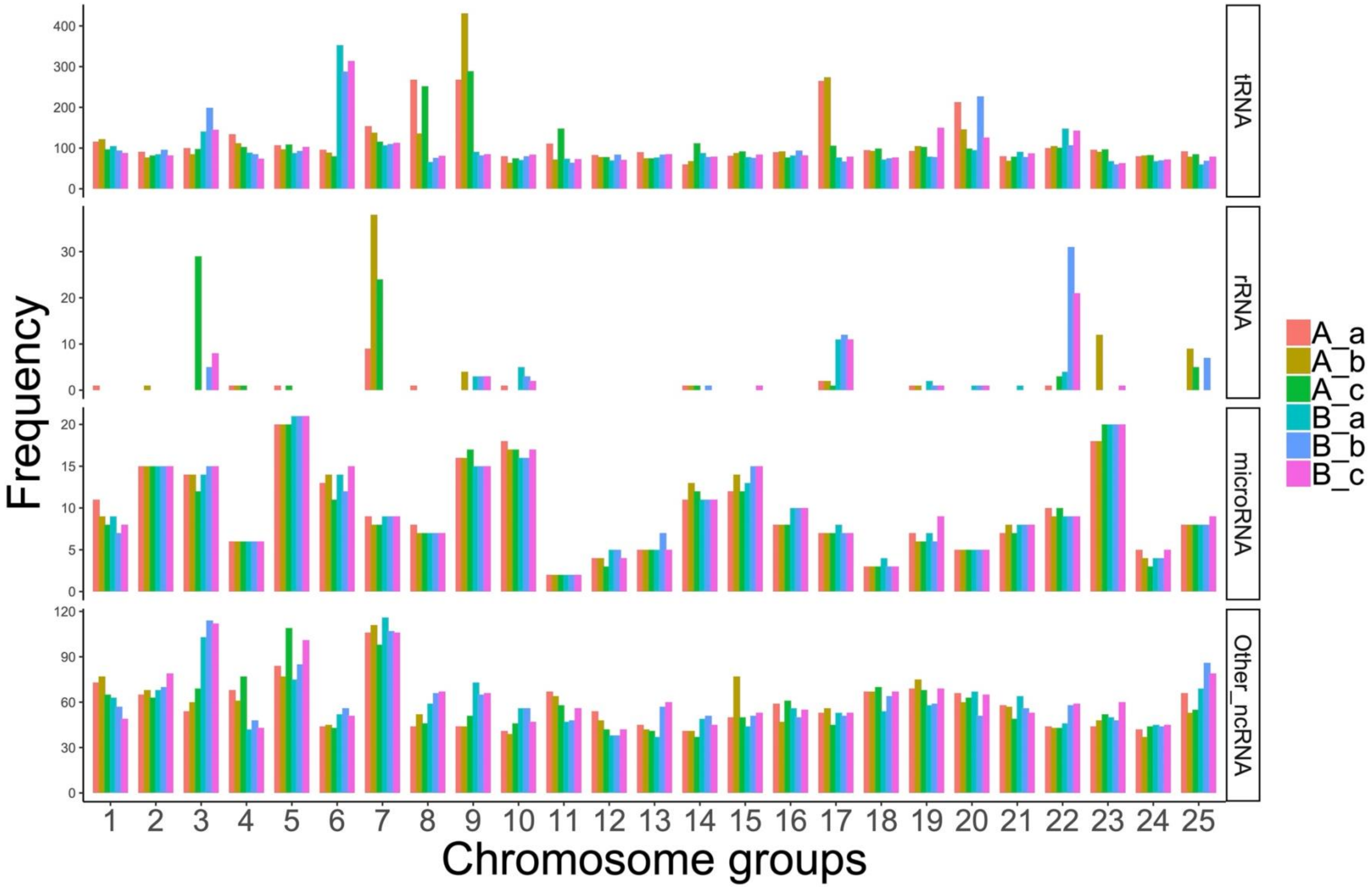

Supplementary Fig. 6: Frequencies of non-coding RNA genes on each chromosome.

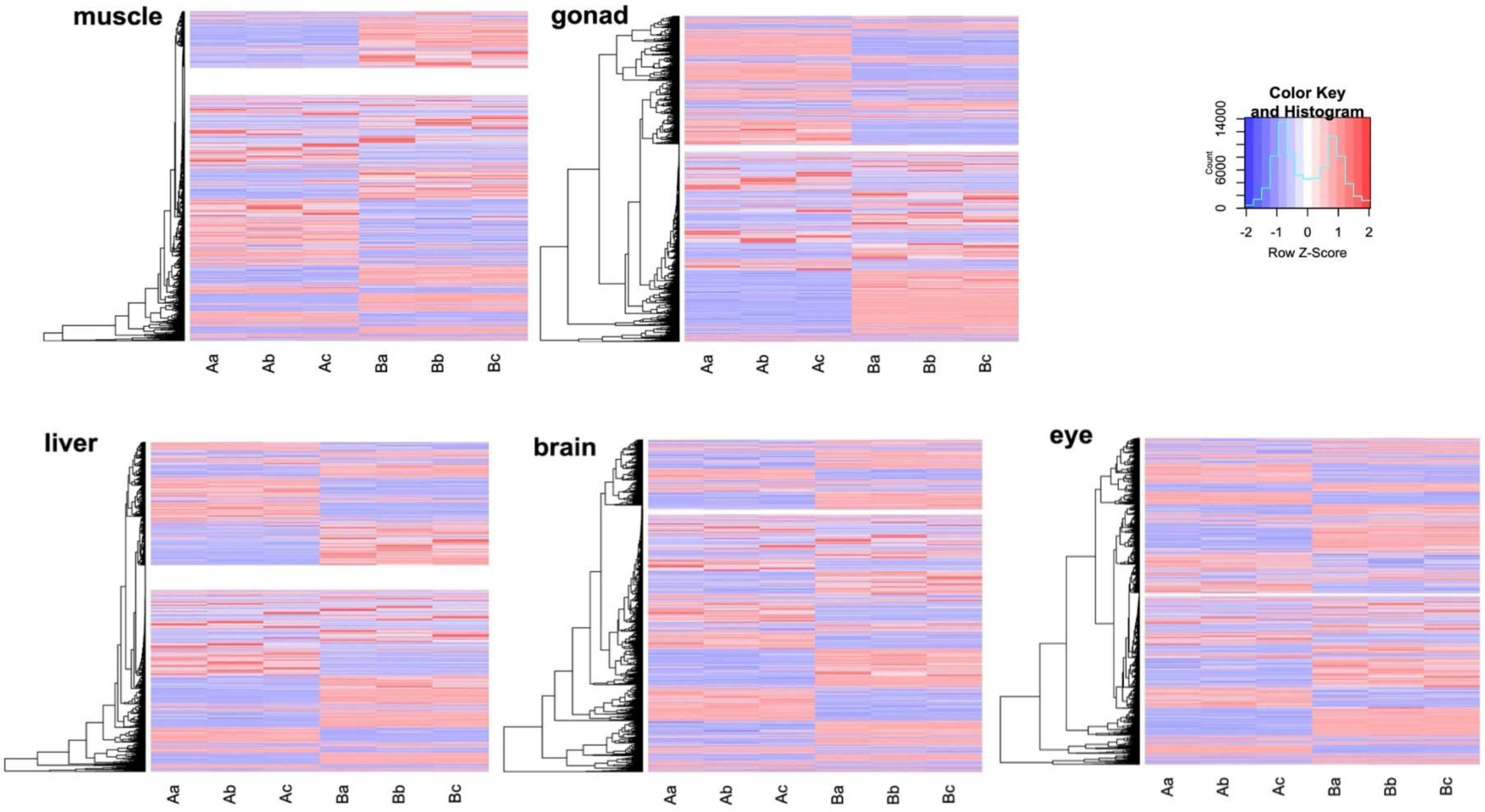

Supplementary Fig. 7: Heatmap showing the expression level of 13,791 homologous genes (rows) across each subgenome and each tissue (columns). The expression level was indexed using $\log_2$(TPM+1), then centered and scaled as Z-Score in the row direction.

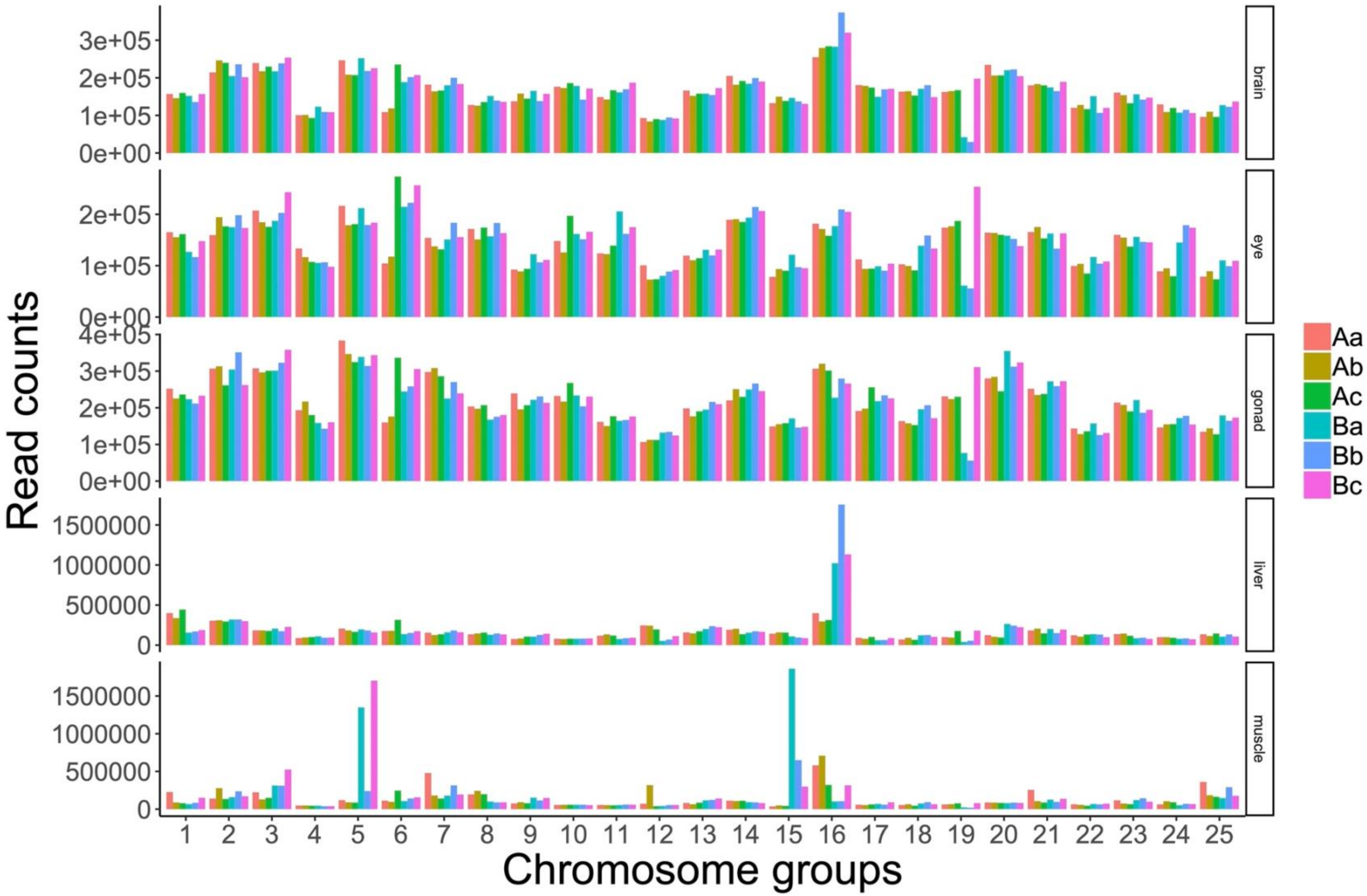

Supplementary Fig. 8: Counts of RNA-seq reads from different organs mapped on the homologous and homeologous genes of the 150 chromosomes of *C. gibelio*. Chromosomes are arranged in 25 syntenic groups, ordered from left to right as subgenomes and haplotypes AaAbAcBaBbBc.

.

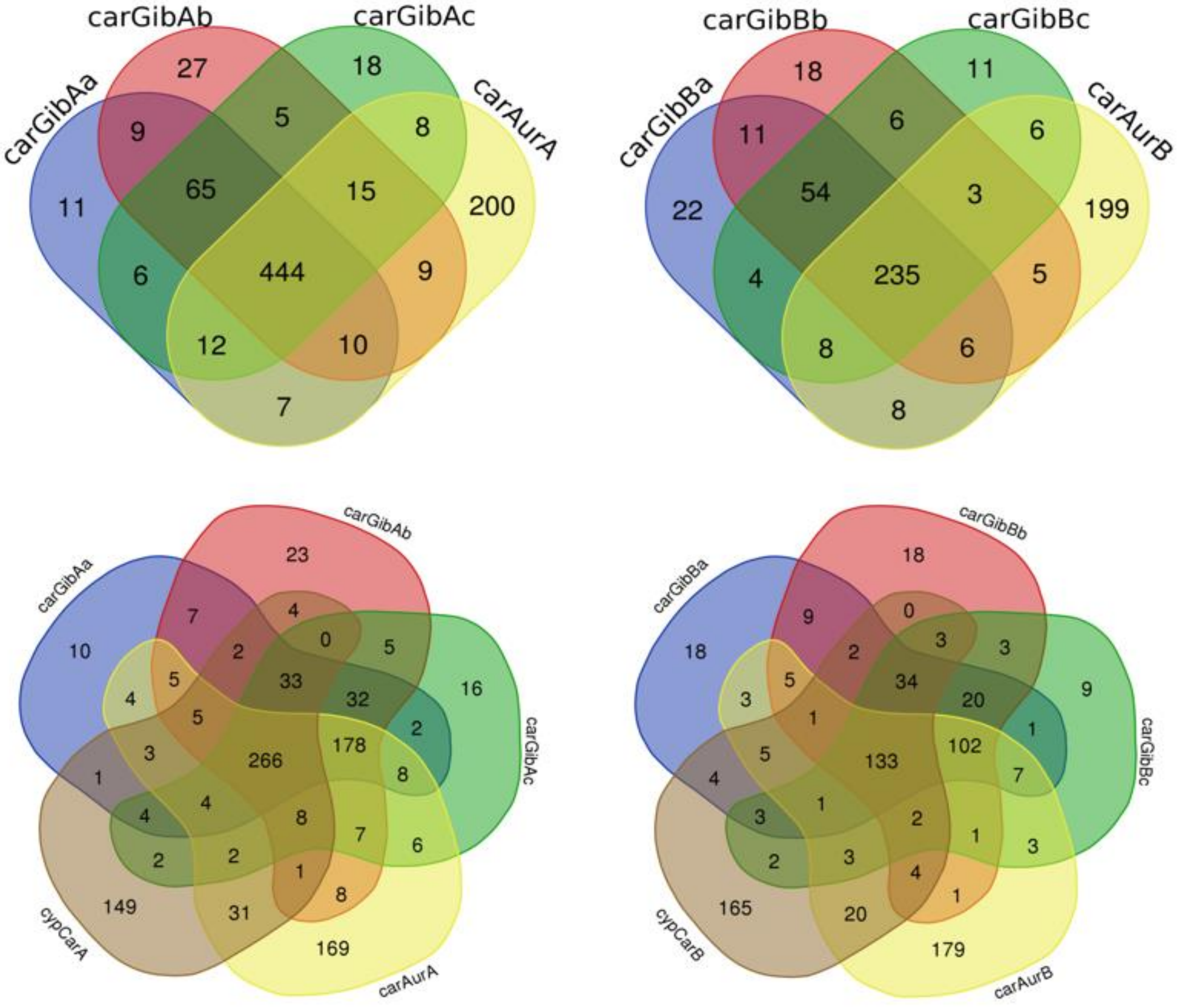

Supplementary Fig. 9: Venn diagrams of subgenome-specific missing BUSCOs based on data as listed in Supplementary Table 7b. The two upper diagrams are comparing *Carassius gibelio* versus *C. auratus*, the two lower diagrams compare *C. gibelio*, *C. auratus* and *Cyprinus carpio*. The diagrams to the left are for subgenome A, and to the right for subgenome B
