# Supplementary material for: Equilibrated evolution of the mixed auto-/allopolyploid haplotype-resolved genome of the invasive hexaploid Prussian carp
Source: Nat Commun. 2022 Jul 14;13:4092. doi: 10.1038/s41467-022-31515-w (PMC9283417; doi:10.1038/s41467-022-31515-w)
Supplement: Supplementary file 2 — Description of Additional Supplementary Files [file 41467_2022_31515_MOESM2_ESM.pdf]

Description of Additional Supplementary Files

File Name: Supplementary Data 1
Description:
1a: HiFi raw data statistics.
1b: Comparison of the haplotype resolved C. gibelio carGib1.2 assembly to available long-read assemblies for Carassius species

File Name: Supplementary Data 2
Description:
Annotation metrics.

File Name: Supplementary Data 3
Description:
3a: Content of genes, repeats and ncRNA, annotated per chromosome. 3b: Repeat classes annotation summary.

File Name: Supplementary Data 4
Description:
4a: Assignment of C. gibelio haplotypes.
4b: Summed evidence from the three tests (see Supplementary Table 4a) used for the assignment of C. gibelio haplotypes.
4c: 4-taxon trees in Newick format. 4d: 12-taxon trees in Newick format.

File Name: Supplementary Data 5
Description:
Sequence difference between haplotype sequences and assembly from another gibel carp (GCA_019843895.1).

File Name: Supplementary Data 6
Description:
Metrics of pairwise Ks values between species/subgenomes.

File Name: Supplementary Data 7
Description:
7a: BUSCO gene content per subgenome.
7b: Missing BUSCOs for Venn diagram.
7c: GO enrichment of missing BUSCOs. The p-value was calculated using the Hypergeometric distribution, which is identical to the corresponding one-tailed version of Fisher's exact test. P-value correction for multiple testing was performed by the "Benjamini Hochberg" method.

File Name: Supplementary Data 8
Description:
8a: Genes lost after autopolyploidy event
8b: GO enrichment. The p-value was calculated using the Hypergeometric distribution, which is identical to the corresponding one-tailed version of Fisher's exact test. Here no p-value correction was performed.
8c: Candidate terms: meiotic, mitotic, chromosome, recombination. The p-value was calculated using the Hypergeometric distribution, which is identical to the corresponding one-tailed version of Fisher's exact test. Here no p-value correction was performed.

File Name: Supplementary Data 9
Description:
Brain expression in logTPM of homologous gene across each subgenome

File Name: Supplementary Data 10
Description:
Eye expression in logTPM of homologous gene across each subgenome

File Name: Supplementary Data 11
Description:
Gonad expression in logTPM of homologous gene across each subgenome

File Name: Supplementary Data 12
Description:
Liver expression in logTPM of homologous gene across each subgenome

File Name: Supplementary Data 13
Description:
Muscle expression in logTPM of homologous gene across each subgenome

File Name: Supplementary Data 14
Description:
Ks value between homologous gene pairs

File Name: Supplementary Data 15
Description:
Expression in Read Counts of each chromosome

File Name: Supplementary Data 16
Description:
Expression in Read Counts of genes on each chromosome
