# Supplementary material for: Equilibrated evolution of the mixed auto-/allopolyploid haplotype-resolved genome of the invasive hexaploid Prussian carp
Source: Nat Commun. 2022 Jul 14;13:4092. doi: 10.1038/s41467-022-31515-w (PMC9283417; doi:10.1038/s41467-022-31515-w)
Supplement: Supplementary file 12 — Reporting Summary [file 41467_2022_31515_MOESM12_ESM.pdf]

nature portfolio

Corresponding author(s): Dunja Lamatsch, Matthias Stöck

Last updated by author(s): 30.05.2022

Reporting Summary

Nature Portfolio wishes to improve the reproducibility of the work that we publish. This form provides structure for consistency and transparency in reporting. For further information on Nature Portfolio policies, see our Editorial Policies and the Editorial Policy Checklist.

Statistics

For all statistical analyses, confirm that the following items are present in the figure legend, table legend, main text, or Methods section.

n/a	Confirmed	
☐	☒	The exact sample size (*n*) for each experimental group/condition, given as a discrete number and unit of measurement
☐	☒	A statement on whether measurements were taken from distinct samples or whether the same sample was measured repeatedly
☐	☒	The statistical test(s) used AND whether they are one- or two-sided<br>*Only common tests should be described solely by name; describe more complex techniques in the Methods section.*
☐	☒	A description of all covariates tested
☐	☒	A description of any assumptions or corrections, such as tests of normality and adjustment for multiple comparisons
☐	☒	A full description of the statistical parameters including central tendency (e.g. means) or other basic estimates (e.g. regression coefficient) AND variation (e.g. standard deviation) or associated estimates of uncertainty (e.g. confidence intervals)
☐	☒	For null hypothesis testing, the test statistic (e.g. *F*, *t*, *r*) with confidence intervals, effect sizes, degrees of freedom and *P* value noted<br>*Give P values as exact values whenever suitable.*
☐	☒	For Bayesian analysis, information on the choice of priors and Markov chain Monte Carlo settings
☐	☒	For hierarchical and complex designs, identification of the appropriate level for tests and full reporting of outcomes
☐	☒	Estimates of effect sizes (e.g. Cohen's *d*, Pearson's *r*), indicating how they were calculated

Our web collection on statistics for biologists contains articles on many of the points above.

Software and code

Policy information about availability of computer code

Data collection	Sequencing data was generated by PacBio HIFI technology or Illumina technology according to the manufacturers protocols of late 2020 / early 2021.
Data analysis	All tools used were open source. Following tools were applied: Hifiasm v0.15.1-r329; Minimap2 v2.22-r1101; bedtools v2.25.0; juicer v1.5.7; 3d-DNA v180922; Juicebox v1.11.08; Minidot v0.3-r179; Last aligner / Last-split v941; Multiz v11.2; Iqtree v2; Repeatmodeler v1.0.8; Repeatmasker version open-4.0.7; Spaln v2.06f;Genewise v2.4.1, Exonerate v2.2.0, Genblasta v1.0.4, fastp v0.21.0, Hisat v2.1.0, StringTie v2.0, Augustus v3.2.3, PAML v4.9, PAL2NAL, MAFFT v7.453, BLAST 2.2.28+

For manuscripts utilizing custom algorithms or software that are central to the research but not yet described in published literature, software must be made available to editors and reviewers. We strongly encourage code deposition in a community repository (e.g. GitHub). See the Nature Portfolio guidelines for submitting code & software for further information.

Data

Policy information about availability of data

All manuscripts must include a data availability statement. This statement should provide the following information, where applicable:

- Accession codes, unique identifiers, or web links for publicly available datasets
- A description of any restrictions on data availability
- For clinical datasets or third party data, please ensure that the statement adheres to our policy

Genome assembly, whole genome sequencing (WGS) data and RNA-seq reads will be available with NCBI accession number PRJNA779620 upon publication and at the Prussian Carp genome browser http://genomes.igb-berlin.de. The annotations are also available at the genome browser. These resources have just been made publicly available with the latest submission.

Field-specific reporting

Please select the one below that is the best fit for your research. If you are not sure, read the appropriate sections before making your selection.

☐ Life sciences ☐ Behavioural & social sciences ☒ Ecological, evolutionary & environmental sciences

For a reference copy of the document with all sections, see nature.com/documents/nr-reporting-summary-flat.pdf

Ecological, evolutionary & environmental sciences study design

All studies must disclose on these points even when the disclosure is negative.

Study description	Genome sequencing and assembly of an ecologically and evolutionary important fish species, no quantitative data were collected
Research sample	The fish used for this study originated from the Olsa river close to Ostrava (Czech Republic) because a C. gibelio neotype was described from this population (Kalous et al. 2012). Organs from a single female were sampled and flash frozen for further processing. Hexaploidy was confirmed by flow cytometry using DAPI following the procedure described (Lamatsch et al. 2000). Use and preparation of the fish were carried out in accordance with approved guidelines under the Austrian law (for details and relevant regulations: Supplementary Note 1).
Sampling strategy	One fish for the reference genome sequence was collected by netting by local fishermen in the pond in the alluvium of Olza River (tributary of Odra River) at Český Těšín; 49°47' 11" N 18°35'24" E; on 5 May 2011.<br>The caught fish was transported alive from the locality of catch in well-aerated containers to the Institute of Animal Physiology and Genetics Czech Academy of Science in Liběchov and placed into concrete ponds situated outdoor in the area of the institute and kept under natural conditions. It was regularly fed by commercial feed. In 2016 ,this fish was transported by Dunja K Lamatsch from IAPG Liběchov to Research Department for Limnology, Mondsee, Austria.
Data collection	one individual was used for DNA and RNA extraction from which all data could be sufficiently generated; the data was collected by EASI-GENOMICS facilities (Uppsala Genome Center, Sweden, and National Genomics Infrastructure, Stockholm, Sweden)
Timing and spatial scale	only one fish was used in this study and supplied enough biological material for all work
Data exclusions	no data were excluded
Reproducibility	only one fish was used in this study and supplied enough biological material for all work
Randomization	only one fish was used in this study and supplied enough biological material for all work
Blinding	only one fish was used in this study and supplied enough biological material for all work

Did the study involve field work? ☐ Yes ☒ No

Reporting for specific materials, systems and methods

We require information from authors about some types of materials, experimental systems and methods used in many studies. Here, indicate whether each material, system or method listed is relevant to your study. If you are not sure if a list item applies to your research, read the appropriate section before selecting a response.

Materials & experimental systems

n/a	Involved in the study
☒	☐ Antibodies
☒	☐ Eukaryotic cell lines
☒	☐ Palaeontology and archaeology
☐	☒ Animals and other organisms
☒	☐ Human research participants
☒	☐ Clinical data
☒	☐ Dual use research of concern

Methods

n/a	Involved in the study
☒	☐ ChIP-seq
☒	☐ Flow cytometry
☒	☐ MRI-based neuroimaging

Animals and other organisms

Policy information about studies involving animals; ARRIVE guidelines recommended for reporting animal research

Laboratory animals	no laboratory animals involved

Wild animals	The used fish is a adult female from the hexaploid biotype from Olza River. It was killed by anasthetic overdose for biopsy of all main organs.
Field-collected samples	One fish for the reference genome sequence was collected by netting by local fishermen in the pond in the alluvium of Olza River (tributary of Odra River) at Český Tešín; 49°47' 11" N 18°35'24" E; on 5 May 2011.<br>The caught fish was transported alive from the locality of catch in well-aerated containers to the Institute of Animal Physiology and Genetics Czech Academy of Science in Liběchov and placed into concrete ponds situated outdoor in the area of the institute and kept under natural conditions. It was regularly fed by commercial feed. In 2016 ,this fish was transported by Dunja K Lamatsch from IAPG Liběchov to Research Department for Limnology, Mondsee, Austria. The fish was kept under 12:12 light:dark cycle at 24°C and fed ad libitum by commercial fish food.
Ethics oversight	The animal was kept and sampled at the ILIM Mondsee in accordance with the regulations of the Austrian Animal Experiment Act (December 28, 2012) (Tierversuchsrechtsänderungsgesetz, part 1, section 1, §1, and point 2), and with the Directive 2010/63/EU of the European Parliament and of the Council of the European Union (September 22, 2010) on the protection of animals used for scientific purposes (chapter 1, article 1, and point 5a). The fish was kept according to regular aquaculture practice, including the provision of appropriate tank size, sufficient rate of waterflow, natural photoperiod, ad libitum food supply, as well as temperatures within the species' thermal tolerance range. This ensured that no pain, suffering, distress or lasting harm was inflicted on the animal. Based on the legislative provisions above, no ethics approval and no IACUC protocol was required for sacrificing the individual after anesthesia with MS222 since it does not incur pain, suffering or distress to the fish, and no formal animal experimentation protocol was required.

Note that full information on the approval of the study protocol must also be provided in the manuscript.
